# GFPT2 drives sunitinib resistance of renal cell carcinoma via enzyme-dependent and -independent manners

**DOI:** 10.7150/ijbs.118985

**Published:** 2026-02-04

**Authors:** Songbo Wang, Jiajun Xing, Xiaoyi Wang, Zengjun Wang, Pengfei Shao, Chenkui Miao

**Affiliations:** 1 Department of Urology, The First Affiliated Hospital of Nanjing Medical University, No. 300 Guangzhou Road, Nanjing, China.; 2 Core Facility Center, The First Affiliated Hospital of Nanjing Medical University, No. 300 Guangzhou Road, Nanjing, China.

**Keywords:** GFPT2, TKI resistance, Renal cell carcinoma, O-GlcNAcylation, non-metabolic function

## Abstract

Intrinsic resistance to sunitinib in advanced renal cell carcinoma (RCC) remains a major barrier to improving patient survival outcomes. However, the molecular mechanisms driving this resistance remain incompletely elucidated. In this study, we first observed elevated glutamine levels in sunitinib-resistant RCC models; notably, glutamine deprivation substantially impaired the growth and proliferation of RCC cells. We further demonstrated that abnormal upregulation of GFPT2—a key enzyme in glutamine metabolism—was associated with reduced sunitinib sensitivity and enhanced drug resistance in RCC. Mechanistically, we uncovered that GFPT2 modulates cellular O-GlcNAcylation levels, which in turn enhances the stability and nuclear translocation of YAP1—ultimately contributing to reduced sunitinib sensitivity. In addition, we also identified an additional non-metabolic role of GFPT2: it directly interacts with the Kelch domain of KEAP1, thereby reducing NRF2 binding to this domain and suppressing NRF2 ubiquitination-dependent degradation. Consequently, this regulatory cascade dysregulates the transcription of downstream antioxidant genes (e.g., HMOX1 and NQO1), ultimately driving NRF2-dependent sunitinib resistance in RCC. Critically, this KEAP1-NRF2 axis-mediated mechanism operates independently of GFPT2's metabolic role in regulating O-GlcNAcylation. Collectively, our findings demonstrate that GFPT2 modulates sunitinib sensitivity and drives drug resistance in RCC via dual mechanisms: a metabolic pathway (O-GlcNAcylation-YAP1) and a non-metabolic pathway (KEAP1-NRF2). Targeting the non-metabolic functions of GFPT2 thus holds promise for enhancing sunitinib sensitivity in RCC while potentially mitigating treatment-related side effects.

## Introduction

Renal cell carcinoma (RCC) is one of the most common malignant tumors in the urinary system. Its incidence rate is continuously on the rise, seriously threatening people's life and health^1^. Early-stage renal cancer patients have a good prognosis after surgical resection of the lesion. However, nearly 30% of patients have distant metastasis at the time of diagnosis. At the same time, 20%- 40% of patients still experience lesion recurrence or metastasis after surgical treatment. Such patients often have a shorter survival period^2^. Since renal cancer is not sensitive to radiotherapy and chemotherapy, molecular targeted drugs such as tyrosine kinase inhibitors (TKI), anti-angiogenic drugs, and immune checkpoint inhibitors have become the first-line preferred treatment strategy for metastatic renal cancer 3-7. Although in the latest National Comprehensive Cancer Network (NCCN) clinical guidelines, immunotherapy combined with immunotherapy or targeted therapy has become the first-line treatment recommendation for advanced metastatic renal cancer. Considering adverse events and economic factors of combination therapy, TKI drugs represented by sunitinib and pazopanib are still widely used in first-line treatment 8, 9. Sunitinib and other TKI drugs have significant efficacy in the treatment of metastatic renal cancer patients in the early stage. However, patients often experience drug resistance progression after 6-15 months of treatment. In the case of failure of first-line TKIs treatment, patients can benefit from second-line treatment drugs such as mTOR inhibitors. But in the end, there is still drug resistance progression 10. Studies have shown that when advanced renal cancer patients with resistance to first-line TKIs develop resistance to second-line mTOR inhibitors and other drugs again, they can still benefit from first-line TKIs drugs, suggesting that TKI drugs still play a very crucial role in the treatment of advanced renal cancer. Multiple studies have shown that the primary resistance of TKI drugs such as sunitinib is mainly related to the increase in tumor angiogenesis and the inability of targeted drugs to inhibit the growth of tumor cells. Acquired resistance is related to various mechanisms, including compensatory activation of angiogenesis, activation of metabolic pathways, non-coding RNA action pathways, plasma and serum drug metabolism pathways, and lysosomal isolation pathways 11, 12.

Approximately 50% of renal cancer patients carry VHL gene mutations 13. Under normal physiological conditions, the pVHL protein (encoded by the VHL gene) negatively regulates the hypoxic response by mediating the ubiquitination and subsequent proteasomal degradation of hypoxia-inducible factors (HIFs)-specifically hypoxia-inducible factor-1α (HIF-1α) and HIF-2α. In the setting of VHL gene deficiency (e.g., mutation or deletion), however, HIF-2α—acting as the primary pathogenic isoform—accumulates aberrantly. This abnormal accumulation drives the persistent activation of HIF-2α downstream target genes, which in turn promotes key oncogenic processes: angiogenesis (e.g., via vascular endothelial growth factor and platelet-derived growth factor), glycolytic metabolic reprogramming (the Warburg effect), and intracellular lipid accumulation. Collectively, these events robustly support tumor initiation and progression.

This VHL-HIF axis mechanism underpins the development of targeted therapies for RCC, such as anti-angiogenic agents (e.g., sunitinib) and HIF-2α inhibitors (e.g., belzutifan). Nevertheless, sustained hyperactivation of the VHL-HIF pathway also contributes to the emergence of drug resistance, necessitating the integration of metabolic intervention and/or immunotherapy to enhance therapeutic efficacy. Additionally, the specific type of VHL mutation correlates with patient prognosis. Notably, in RCC with wild-type VHL (VHL-WT RCC), alternative HIF activation pathways independent of VHL may exist, underscoring the complexity of the molecular mechanisms driving this disease 14, 15.

The latest research shows that metabolism-related pathways are also involved in the regulation of sensitivity or drug resistance of renal cancer to TKIs. Some researchers used liquid chromatography-mass spectrometry to conduct metabolomic analysis on sunitinib-resistant renal cancer cell lines. It was found that metabolites such as glutamine, glutamate, alpha-ketoglutaric acid, and fructose-6-phosphate were significantly up-regulated in drug-resistant cell lines, suggesting that the abnormal activation of related metabolic pathways may be closely related to the process of sunitinib resistance 16-18. But so far, the key role of metabolic signaling pathways in the process of sunitinib resistance in renal cancer and the upstream and downstream regulatory mechanisms are still blank 17, 19.

A pivotal observation is the noteworthy involvement of amino acid metabolism, particularly, in the pathophysiology of renal cancer 18. Despite the acknowledged significance of glutamine in various solid tumors such as prostate cancer, breast cancer, and liver cancer, the precise mechanistic framework governing its regulatory role in renal cancer remains elusive 20, 21. Notably, preceding studies have identified elevated levels of glutamine in models resistant to Sunitinib, prompting an in-depth exploration to elucidate the specific mechanistic underpinnings of this phenomenon. This investigation aims to contribute to the comprehension of the intricate interplay between glutamine metabolism and Sunitinib resistance in renal cancer.

Cancer cells frequently exhibit heightened activity in the hexosamine biosynthesis pathway (HBP), with glutamine-fructose-6-phosphate transaminase 2 (GFPT2) acting as the rate-limiting enzyme 22, 23. GFPT2 encodes a protein responsible for converting glutamine to fructose-6-phosphate, thereby regulating the rate of the HBP. This enzyme is crucial for glucose synthesis and has been linked to the altered metabolic landscape characteristic of cancer. Recent studies have demonstrated that GFPT2 is associated with tumor progression in various types of cancer 24-26.

Metabolic reprogramming is a hallmark of cancer, characterized by altered glycolytic pathways that fulfill the increased energy production, biosynthesis, and growth demands within tumors 27. A pivotal component of this metabolic process is the hexosamine biosynthetic pathway (HBP). As glucose uptake rises, a portion (2-5%) is redirected into the HBP, leading to the synthesis of uridine 5-diphospho-N-acetylglucosamine (UDP-GlcNAc)—a fundamental substrate for protein glycosylation. O-GlcNAcylation, a specific form of protein glycosylation mediated by O-linked N-acetylglucosamine transferase (OGT), involves the attachment of O-GlcNAc to serine and threonine residues in various cytoplasmic, nuclear, and mitochondrial proteins 28, 29. This modification process is tightly regulated by the concentration of UDP-GlcNAc available 30. Dysregulation of O-GlcNAcylation has been associated with cancer promotion through gene regulatory mechanisms, highlighting its significant role in cancer biology 31.

Nuclear factor erythroid 2-related factor 2 (NRF2) is subjected to ubiquitination-mediated degradation by Kelch-like ECH-associated protein 1 (KEAP1) under physiological conditions. Upon exposure to electrophilic reagents or oxidative stress, NRF2 dissociates from KEAP1, translocates to the nucleus, and functions as a transcription factor. Within this nuclear environment, NRF2 promotes the transcription of downstream antioxidant genes, including HMOX1, AKR1C1, and NQO1, among others.^32^. This coordinated transcriptional response plays a crucial role in cellular defense against oxidative stress, thereby enhancing the survival of tumor cells in hostile environments. The literature has implicated NRF2 in mediating resistance to anticancer drugs, such as Enzaruane and 5-fluorouracil, induced by anti-oxidation mechanisms. Despite its recognized importance as a potential therapeutic target, the KEAP1/NRF2 axis remains relatively underexplored in the context of kidney cancer 33.

Our empirical studies demonstrate that GFPT2 competes directly for the NRF2-binding site on KEAP1. This competitive interaction blocks KEAP1-mediated degradation of NRF2, and importantly, occurs independently of the O-GlcNAcylation pathway. Collectively, these findings corroborate the notion that GFPT2 plays a pivotal role in driving resistance to Sunitinib through both metabolic and non-metabolic mechanisms.

## Results

### Glutamine regulates the sensitivity of sunitinib in renal cell carcinoma

**Figure 1:**
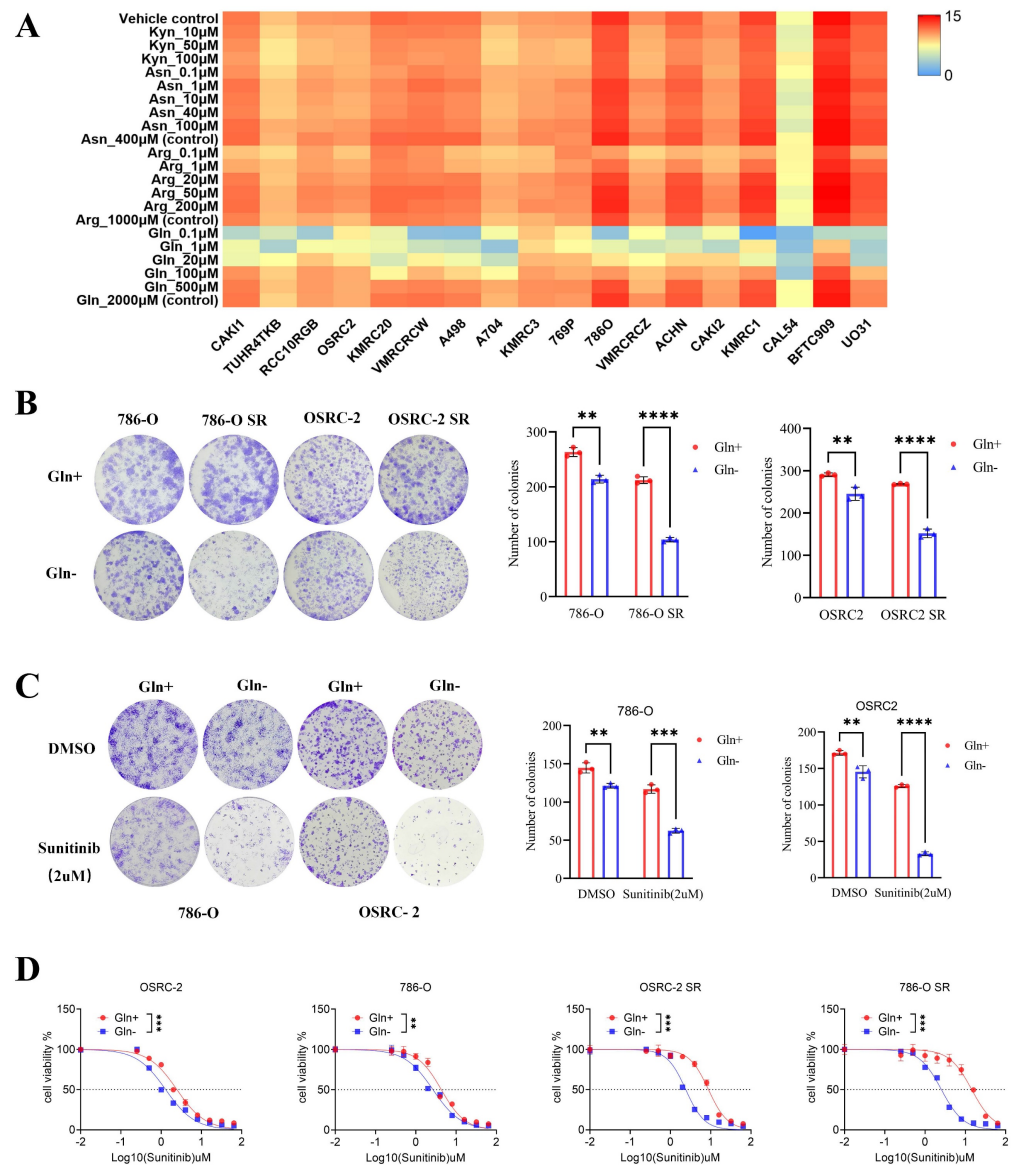
** Glutamine regulates the sensitivity of sunitinib in renal cell carcinoma.** (A) Sensitivity of renal cancer cells (from the CCLE database) to kynurenic acid, aspartic acid, arginine, and glutamine across a concentration gradient (0 to conventional levels). (B) Colony formation assays assessed proliferation of 786-O, 786-O SR, OSRC-2, and OSRC-2 SR cells cultured with or without glutamine. Colonies were quantified using ImageJ. Results are derived from three independent experiments. P values were determined by two-tailed t test. **P < 0.01, ****P < 0.0001. (C) Colony formation assays measured growth of 786-O and OSRC-2 cells (cultured with or without glutamine) after sunitinib treatment. Colonies were quantified using ImageJ. Results are derived from three independent experiments. P values were determined by two-tailed t test; **P < 0.01, ***P < 0.001, ****P < 0.0001. (D) 786-O, 786-O SR, OSRC-2, and OSRC-2 SR cells (cultured with or without glutamine) were treated with serial doses of sunitinib for 24 h, then subjected to CCK-8 assay.

Amino acid metabolism is well recognized for its critical roles in cancer cell proliferation, metastasis, and drug resistance. To investigate the dependence of RCC cells on specific amino acids, we analyzed the responses of all RCC cell lines in the Cancer Cell Line Encyclopedia (CCLE) database to gradual depletion of four amino acids—kynurenic acid, aspartic acid, arginine, and glutamine—across a concentration range from 0 to physiological levels. Cell proliferation assays demonstrated that glutamine depletion significantly impaired RCC cell growth and proliferation (Fig. 1A), whereas depletion of kynurenic acid, aspartic acid, or arginine at low concentrations exerted no appreciable effect on RCC cell proliferation. This observation is consistent with the established role of glutamine in supporting the metabolic reprogramming, wherein it contributes to energy metabolism, tumor progression, and drug resistance.

To explore the association between glutamine and sunitinib resistance, we first established two sunitinib-resistant RCC cell lines (786-O SR and OSRC-2 SR) through prolonged exposure to stepwise increasing concentrations of sunitinib (Fig. S1A-B). We then depleted glutamine from the culture medium of these resistant lines and their parental counterparts, followed by colony formation assays to assess cell proliferation. Results showed that the proliferation of sunitinib-resistant cells was inhibited to a greater extent than that of parental cells under glutamine-depleted conditions (Fig. 1B), indicating that sunitinib-resistant RCC cells exhibit enhanced reliance on exogenous glutamine.

To further validate the regulatory role of glutamine in sunitinib resistance, we performed in vitro CCK-8 and colony formation assays. Specifically, when sunitinib-treated RCC cells were subjected to glutamine depletion, this intervention resulted in a significant reduction in cell viability, a decrease in sunitinib's half-maximal inhibitory concentration (IC₅₀) value (reflecting enhanced drug sensitivity), and a marked reduction in both the number and size of colonies (Fig. 1C-D). Collectively, these findings demonstrate that glutamine is essential for sustaining sunitinib resistance in RCC. Depletion of glutamine restores sunitinib sensitivity by impairing the viability and proliferative capacity of RCC cells, highlighting glutamine as a critical mediator of sunitinib resistance in this disease.

### GFPT2 plays a key role in modulating the sensitivity of RCC to sunitinib

**Figure 2:**
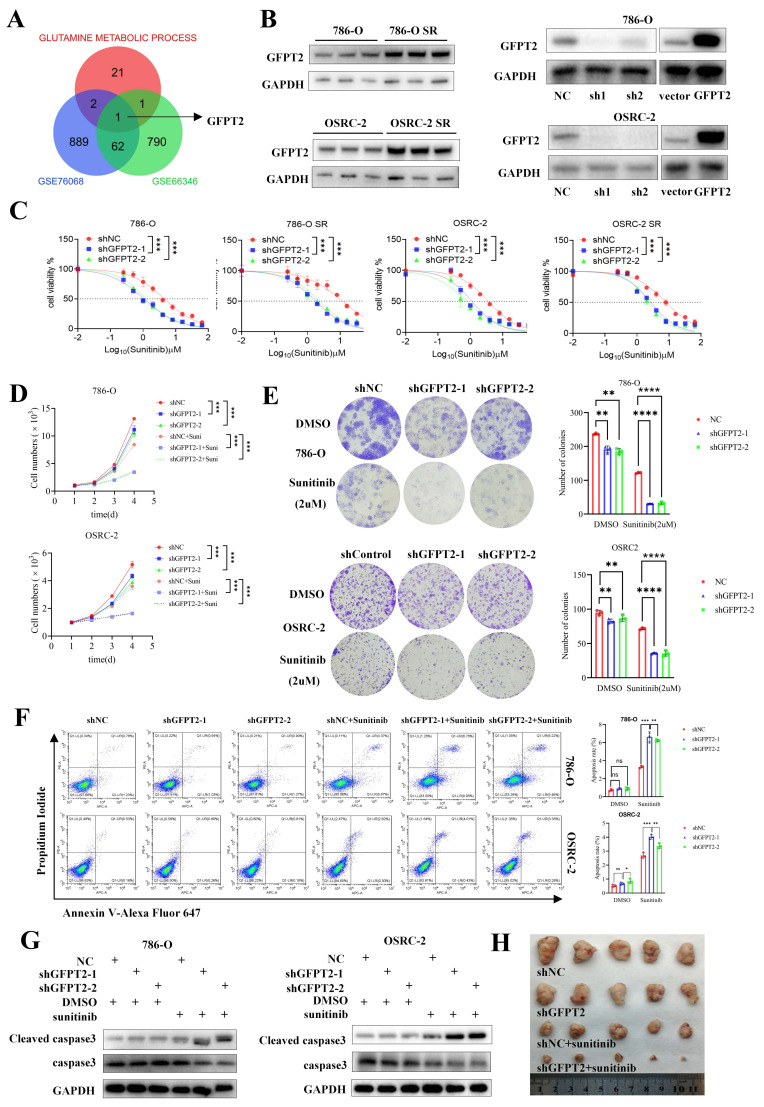
** GFPT2 plays a key role in modulating the sensitivity of RCC to sunitinib.** (A) Intersection of genes from GSE76068, GSE66346 datasets and a gene set associated with glutamine metabolic process. (B) GFPT2 protein levels in 786-O, OSRC-2, sunitinib-resistant 786-O (786-O SR), and sunitinib-resistant OSRC-2 (OSRC-2 SR) cells were detected by Western blotting. 786-O and OSRC-2 cells were transfected with indicated constructs for 72 h, subjected to puromycin selection, and then harvested for Western blot analysis, sh1: shGFPT2-1, sh2: shGFPT2-2. (C) 786-O, OSRC-2, 786-O SR, and OSRC-2 SR cells were transfected with indicated constructs for 72 h. After puromycin selection, cells were treated with serial doses of sunitinib for 24 h, and CCK-8 assays were used to determine sunitinib IC50 values for each group. (D) 786-O, OSRC-2, 786-O SR, and OSRC-2 SR cells were transfected with indicated constructs for 72 h. After puromycin selection, cells were treated with or without sunitinib (2 μM) for 96 h, and CCK-8 assays assessed cell viability. P values were calculated by two-tailed t test. ***P < 0.001. (E) Colony formation assays evaluated the growth of 786-O, OSRC-2, 786-O sh-GFPT2-1, OSRC-2 sh-GFPT2-1, 786-O sh-GFPT2-2, and OSRC-2 sh-GFPT2-2 cells after sunitinib (2 μM) treatment. Results are derived from three independent experiments. P values were determined by two-tailed t test. **P < 0.01, ****P < 0.0001. (F-G) 786-O and OSRC-2 cells were transfected with indicated constructs for 48 h, followed by 24 h of puromycin selection. Cells were then treated with or without sunitinib (2 μM) for an additional 24 h and subjected to (F) Annexin V-Alexa Fluor 647/7-AAD apoptosis assays, or (G) Western blot analysis. Data are presented as mean ± SEM; ns, not significant; **P < 0.01; ***P < 0.001. (H) OSRC-2 cells were transfected with indicated constructs, and subcutaneously injected into nude mice. Mice were treated with or without sunitinib (oral gavage, 25 mg/kg, once daily for 8 days). Representative tumor images are shown in panel H. Data are presented as mean ± SEM (n = 5 mice per group); **P < 0.01; ***P < 0.001.

To further investigate the association between glutamine metabolism and sunitinib resistance in RCC, we acquired two sunitinib resistance-related kidney cancer datasets from the Gene Expression Omnibus (GEO) database, along with a gene set involved in glutamine metabolism. Bioinformatic analyses revealed that glutamine metabolism-associated gene GFPT2 exhibited a potential correlation with sunitinib resistance in RCC (Fig. 2A).

Subsequently, we examined the expression pattern of GFPT2 in RCC. As shown in Fig. S2B, GFPT2 mRNA expression was significantly upregulated in RCC tissues relative to normal kidney tissues, based on data from The Cancer Genome Atlas (TCGA). This upregulation was further validated in RCC cell lines obtained from the CCLE database (Fig. S2A). Further clinicopathological correlation analysis demonstrated a significant positive association between GFPT2 mRNA expression and key clinicopathological parameters, including T stage and pathological TNM stage (Fig. S2D). Survival analysis indicated that high GFPT2 expression was independently associated with shorter overall survival in patients with RCC (Fig. S2C).

We also validated GFPT2 expression at both the mRNA and protein levels in seven RCC cell lines and ten pairs of RCC tissues. As presented in Fig. S2E-G, GFPT2 expression was significantly higher in RCC cell lines than in the normal renal epithelial cell line HK-2. Additionally, a marked upregulation of GFPT2 was observed in RCC tissues relative to their adjacent non-cancerous counterparts. Notably, GFPT2 expression was also elevated in sunitinib-resistant RCC cells compared to their parental cell lines (Fig. 2B). Collectively, these results confirm that GFPT2 is aberrantly overexpressed in RCC and correlates with poor prognosis in patients with this disease.

To delineate the functional role of GFPT2 in sunitinib resistance, we performed GFPT2 overexpression and knockdown experiments in both 786-O and OSRC-2 RCC cell lines (Fig. 2B). Functional assays showed that GFPT2 knockdown significantly enhanced the sensitivity of both sunitinib-resistant RCC cells and their parental cells to sunitinib, whereas GFPT2 overexpression reduced sunitinib sensitivity (Fig. 2C). Consistent results were obtained from CCK-8 proliferation assays (Fig. 2D) and colony formation assays (Fig. 2E), collectively demonstrating that GFPT2 promotes sunitinib resistance in RCC.

In addition, flow cytometry assays revealed a significant increase in apoptosis in 786-O and OSRC-2 cells following GFPT2 knockdown, whereas GFPT2 overexpression decreased sunitinib-induced apoptosis (Fig. 2F and Fig. S3E). Caspase-3 activity assays and western blotting analysis further confirmed that, compared with sunitinib treatment alone, GFPT2 knockdown increased the level of cleaved caspase-3 and enhanced caspase-3 activity in sunitinib-treated 786-O and OSRC-2 cells (Fig. 2G and Fig. S3A).

Moreover, in vivo studies demonstrated that GFPT2 downregulation enhanced sunitinib sensitivity in RCC xenograft models (Fig. 2H and Fig. S3B-C), whereas GFPT2 overexpression exerted the opposite effect in both in vitro and in vivo contexts (Fig. S3D-I). Taken together, these findings establish that GFPT2 plays a pivotal role in regulating the sensitivity of RCC cells to sunitinib.

### GFPT2 contributes to sunitinib resistance of RCC though enhancing YAP1 protein stability and nucleus translocation

**Figure 3:**
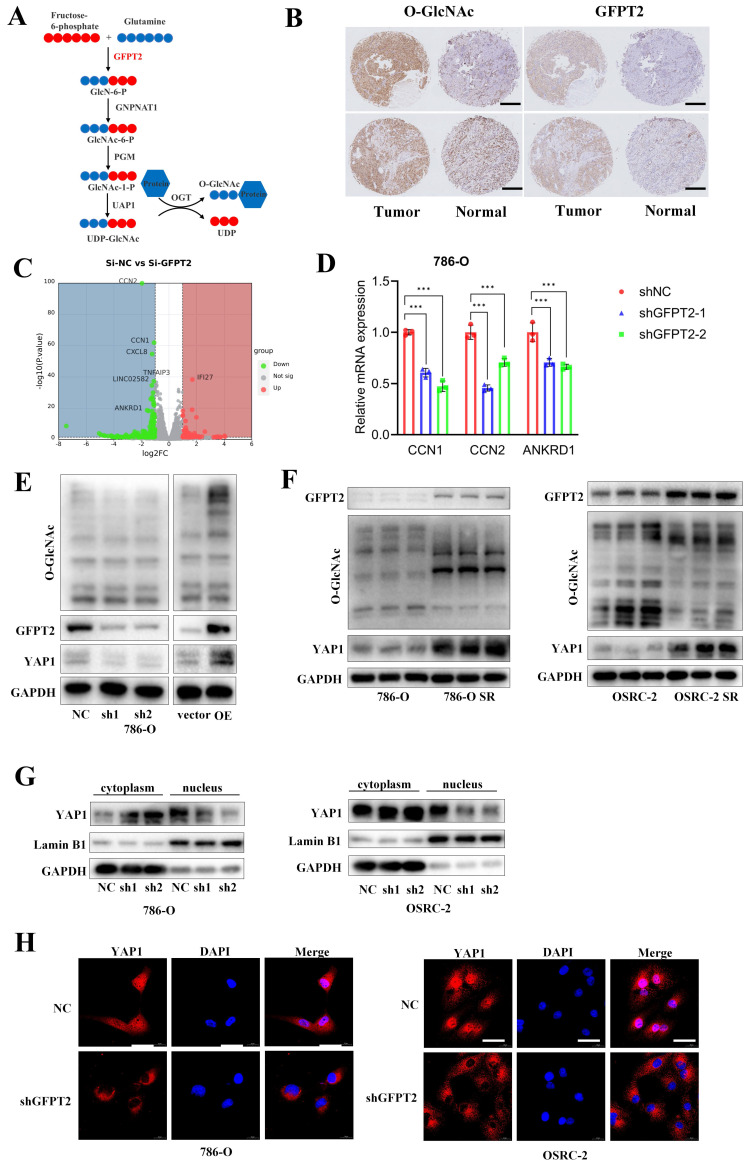
** GFPT2 contributes to sunitinib resistance of RCC though enhancing YAP1 protein stability and nucleus translocation.** (A) Schematic diagram illustrating that GFPT2 functions as a key regulatory enzyme for O-GlcNAcylation. (B) Tissue microarrays containing renal cancer and adjacent non-tumor tissues were subjected to immunohistochemical (IHC) staining for O-GlcNAc and GFPT2. Representative IHC images are shown in panel B. Scale bars, 400 μm. (C) Transcriptome profiling of 786-O cells transfected with siNC or si-GFPT2 for 48 h. (D) qRT-PCR validation of YAP1 target genes (CCN1, CCN2, and ANKRD1) with differential expression identified by RNA-seq. Results are derived from three independent experiments. P values were determined by two-tailed t test. ***P<0.001. (E) Western blot analysis of GFPT2, YAP1, and O-GlcNAc protein levels in 786-O and OSRC-2 cells transfected with indicated constructs for 48 h. (F) Western blot analysis of GFPT2, YAP1, and O-GlcNAc protein levels in 786-O SR and OSRC-2 SR cells. (G) Western blot analysis of YAP1 distribution in the cytoplasm and nucleus of 786-O and OSRC-2 cells with GFPT2 knockdown. (H) Representative immunofluorescence images showing YAP1 subcellular localization in GFPT2-knockdown or negative control 786-O and OSRC-2 cell lines. Scale bars, 10 μm.

Recent studies have established that GFPT2 serves as a critical rate-limiting enzyme in the hexosamine biosynthetic pathway (HBP), where it regulates the flux of UDP-GlcNAc (the key substrate for O-GlcNAcylation) and thereby modulates global glycosylation levels (Fig. 3A). To validate the association between GFPT2 and O-GlcNAcylation in RCC, we performed IHC staining on 103 pairs of RCC tumor tissues and their adjacent non-tumor tissues. Consistent with previous reports, our results confirmed a significant positive correlation between O-GlcNAcylation levels and GFPT2 expression (Fig. 3B).

To further delineate the molecular mechanism by which GFPT2 regulates sunitinib sensitivity in RCC, we transfected 786-O cells with GFPT2-specific small interfering RNAs (siRNAs) or non-targeting control siRNA, followed by RNA sequencing (RNA-seq) analysis (Fig. 3C). Differentially expressed genes (DEGs) identified from RNA-seq were subsequently validated by quantitative real-time PCR (qRT-PCR). As anticipated, qRT-PCR results showed that the mRNA levels of CCN1, CCN2, and ANKRD1—well-characterized downstream effectors of the YAP1 signaling pathway—were significantly reduced in GFPT2-knockdown cells (Fig. 3D). These data suggested that GFPT2 may modulate the YAP1 signaling pathway in RCC.

Notably, YAP1 is well-documented to play a pivotal role in promoting cell proliferation, metastasis, and sunitinib resistance in RCC 34,35. Additionally, YAP1 has been reported to serve as a substrate for O-GlcNAcylation. Given that GFPT2 is the rate-limiting enzyme governing O-GlcNAcylation, we next detected the protein expression levels of YAP1 and global O-GlcNAcylation in GFPT2-knockdown and GFPT2-overexpressing RCC cells. Following GFPT2 knockdown, we observed a significant reduction in global O-GlcNAcylation, which was accompanied by a marked decrease in YAP1 protein expression. Conversely, GFPT2 overexpression led to increased global O-GlcNAcylation and a corresponding upregulation of YAP1 protein levels (Fig. 3E). Furthermore, western blotting analysis confirmed that both YAP1 protein expression and global O-GlcNAcylation levels were significantly higher in sunitinib-resistant RCC cells than in their parental cells (Fig. 3F).

O-GlcNAcylation of target proteins is known to regulate their subcellular localization, including nuclear translocation. To evaluate the effect of GFPT2 on YAP1 localization, we performed nuclear-cytoplasmic fractionation assays. Results showed that GFPT2 knockdown significantly reduced the nuclear expression of YAP1 (Fig. 3G), which was consistent with the YAP1 subcellular localization pattern detected by IF staining (Fig. 3H).

Collectively, our findings demonstrate that GFPT2 promotes sunitinib resistance in RCC by enhancing YAP1 protein stability and facilitating its nuclear translocation.

### GFPT2 partially regulates sunitinib resistance of RCC in enzyme-dependent manners

GFPT2 is well recognized as a key enzyme in the hexosamine biosynthetic pathway (HBP). To address whether the canonical metabolic enzymatic activity of GFPT2 is essential for its regulation of sunitinib sensitivity in RCC, we designed YAP1-specific siRNAs and a GFPT2-knockdown lentivirus. Subsequently, we transfected YAP1 siRNAs into 786-O and OSRC-2 cells with stable endogenous GFPT2 knockdown.

**Figure 4:**
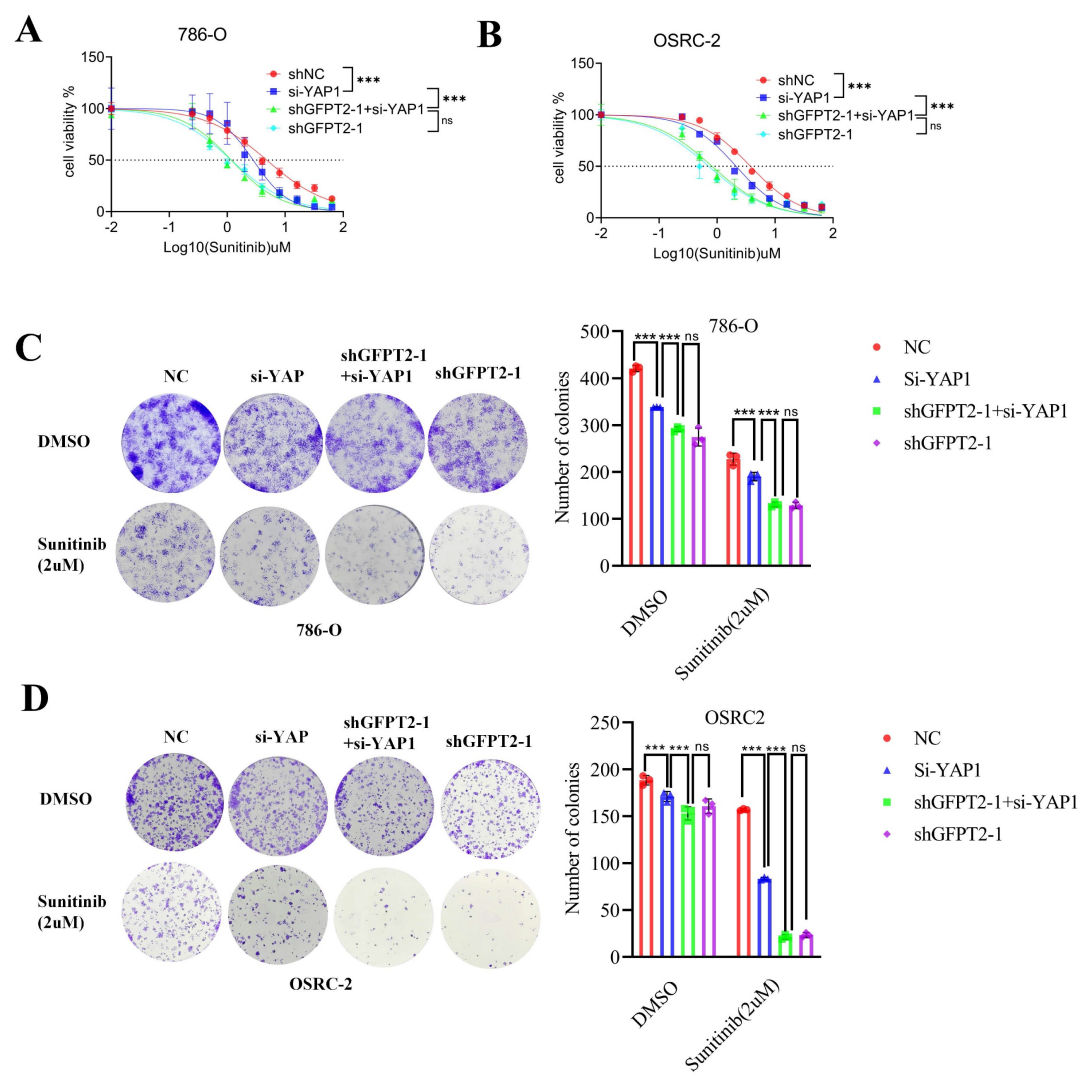
** GFPT2 partially regulates sunitinib resistance of RCC in enzyme-dependent manners.** (A-B). 786-O (A) and OSRC-2 (B) cells were transfected with indicated constructs for 48h. After puromycin selection, these cells were treated with a serial dose of sunitinib for 24h and subjected to CCK-8 assay. (C-D). Colony formation assays were employed to assess the proliferation capacity of 786-O (C) and OSRC-2 (D) transfected with indicated constructs. Colony number was quantified using ImageJ software. Results are derived from three independent experiments. P values were determined by two-tailed t test. ns, not significant and ***P < 0.001.

Our results showed that YAP1 interference significantly enhanced the sensitivity of RCC cells to sunitinib. Notably, GFPT2 knockdown still exerted a more potent effect in enhancing sunitinib sensitivity compared to YAP1 interference alone (Fig. 4A-B). Furthermore, in colony formation assays, YAP1 interference alone significantly enhanced the sensitivity of RCC cells to sunitinib; however, this effect was notably weaker than that induced by GFPT2 knockdown (Fig. 4C-D).

These data demonstrate that GFPT2 regulates sunitinib sensitivity in RCC not only through its canonical enzymatic activity but also via an as-yet-unidentified enzyme-independent mechanism.

### GFPT2-mediated NRF2 stabilization promoted RCC sunitinib resistance

**Figure 5:**
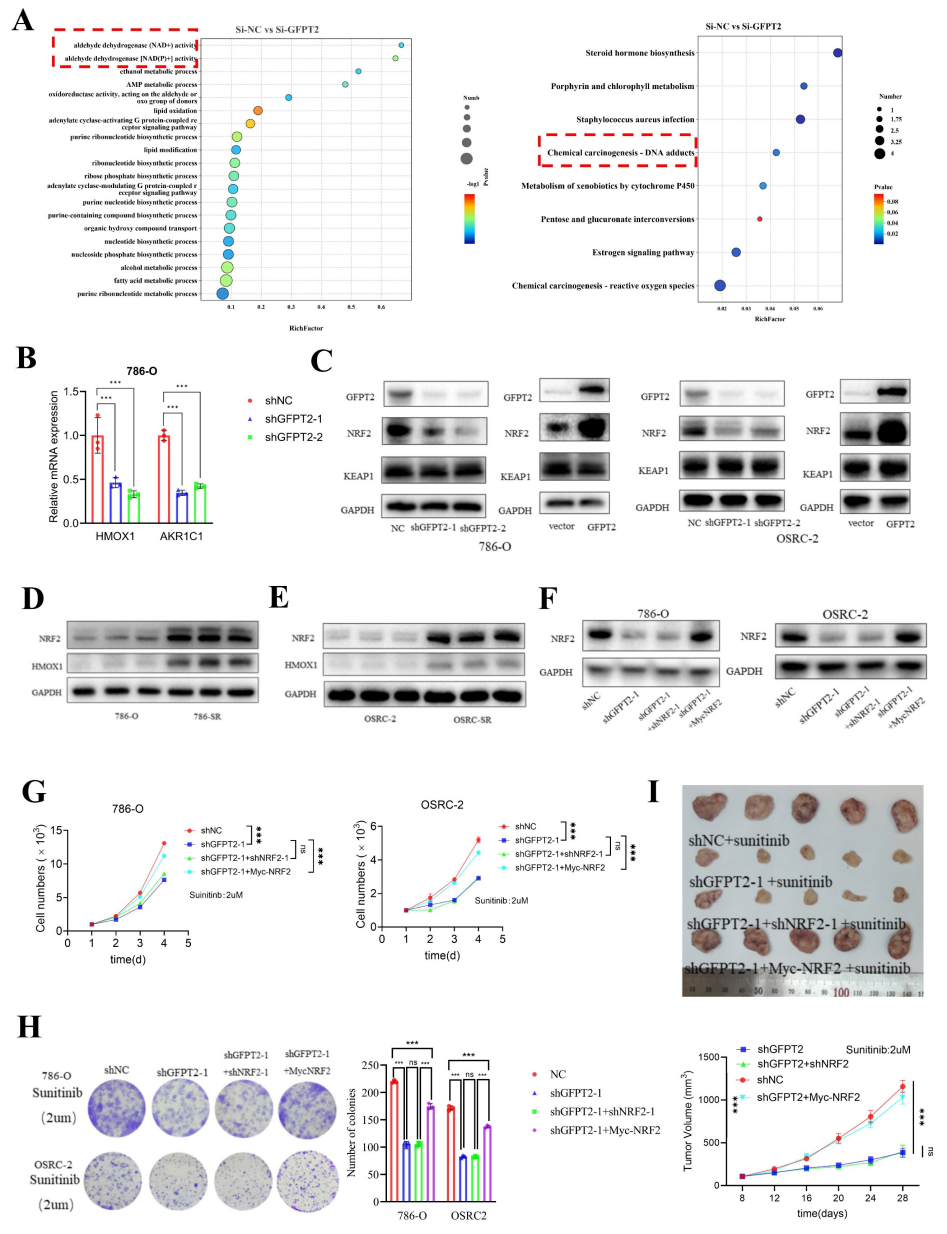
** GFPT2-mediated NRF2 stabilization promoted RCC sunitinib resistance.** (A) GO and KEGG enrichment analyses of RNA-seq data from 786-O cells with GFPT2 knockdown. P values are indicated. (B) qRT-PCR analysis of ROS-related pathway gene expression in cells transfected with negative control shRNA or GFPT2-targeting shRNA. (C) Western blot analysis of GFPT2, KEAP1, and NRF2 protein levels in 786-O and OSRC-2 cells transfected with indicated constructs for 48 h. (D-E) Western blot analysis of NRF2 and HMOX1 protein levels in 786-O, OSRC-2, 786-O SR, and OSRC-2 SR cells. (F) Western blot analysis of NRF2 protein levels in 786-O and OSRC-2 cells transfected with indicated constructs. (G) 786-O and OSRC-2 cells (NC, shGFPT2, shGFPT2+shNRF2, shGFPT2+Myc-NRF2) were treated with sunitinib for the indicated days. Cell proliferation was assessed by CCK-8 assay. P values were calculated by two-tailed t test. ns, not significant and ***P < 0.001. (H) Colony formation assays evaluated the growth of 786-O and OSRC-2 cells (NC, shGFPT2, shGFPT2+shNRF2, shGFPT2+Myc-NRF2) after sunitinib (2 μM) treatment. Results are derived from three independent experiments. P values were determined by two-tailed t test. ns, not significant and ***P < 0.001. (I) OSRC-2 cells were transfected with indicated constructs for 72 h, subjected to puromycin selection, and subcutaneously injected into nude mice. Mice were treated with or without sunitinib (oral gavage, 25 mg/kg, once daily for 8 days). Representative tumor images are shown in panel I. Data are presented as mean ± SEM (n = 5 mice per group); ns, not significant and ***P< 0.001.

To investigate the enzyme-independent mechanism by which GFPT2 regulates sunitinib resistance, we conducted transcriptome analysis using previously generated data. Kyoto Encyclopedia of Genes and Genomes (KEGG) and Gene Ontology (GO) enrichment analyses of the RNA-seq data indicated that GFPT2 silencing modulated several signaling pathways, including those related to NADP+ activity and reactive oxygen species (ROS) generation (Fig. 5A). These findings are particularly intriguing, as ROS are known to contribute to multiple forms of tumor drug resistance. To validate these enrichment results, we performed qRT-PCR analysis in GFPT2-depleted cells. The results showed that oxidative stress-related genes—such as HMOX1 and AKR1C1—were significantly downregulated in GFPT2-knockdown cells (Fig. 5B). Subsequently, we analyzed the expression pattern of HMOX1 in the TCGA RCC cohort. As illustrated in Fig. S4A, the mRNA expression of HMOX1 was significantly higher in RCC tissues than in normal kidney tissues.

According to previous reports, NRF2 regulates antioxidant genes, which in turn promotes ROS elimination and reduces inflammation. Activation of the NRF2 pathway enhances cell survival under oxidative stress or xenobiotic insult 38-40. Importantly, many NRF2 target genes—including HMOX1, AKR1C1, drug-metabolizing enzymes, antioxidant enzymes, and drug transporters—play a crucial role in mediating chemoresistance 41. Based on our findings and prior reports, we hypothesized that NRF2 may regulate the sensitivity of RCC cells to sunitinib. Thus, to investigate the role of NRF2 in sunitinib resistance, we performed NRF2 overexpression and knockdown in sunitinib-resistant RCC cell lines and their parental counterparts, respectively. These results indicated that NRF2 knockdown enhanced the sensitivity of RCC cells to sunitinib both in vitro and in vivo (Fig. S4B-E).

Next, we aimed to further job to explore the mechanism of how GFPT2 regulates sensitivity to sunitinib in RCC via regulating NRF2. As shown in Fig. 5C, NRF2 protein expression was upregulated in 786-O and OSRC-2 cells with GFPT2 overexpression, and downregulated in those with GFPT2 knockdown. We performed IHC staining on RCC tissue microarrays to analyze the correlation between GFPT2 and NRF2 expression, which revealed a positive correlation between their expression levels in RCC tissues (Fig. S4F). These results suggested that GFPT2 regulates NRF2 expression. In addition, both NRF2 and HMOX1 expression were upregulated in sunitinib-resistant cells compared to their parental cells (Fig. 5D-E). We transfected a Myc-NRF2 plasmid into GFPT2-knockdown 786-O and OSRC-2 cells (Fig. 5F). To confirm the impact of NRF2 overexpression on sunitinib sensitivity, we performed CCK-8 assays, colony formation assays, and xenotransplantation assays. The results demonstrated that NRF2 overexpression resulted in incomplete restoration of sunitinib resistance in GFPT2-knockdown RCC cells (Fig. 5G-I). Collectively, these findings indicate that GFPT2-mediated NRF2 stabilization promotes sunitinib resistance in RCC.

### GFPT2 impeded the degradation of NRF2

**Figure 6:**
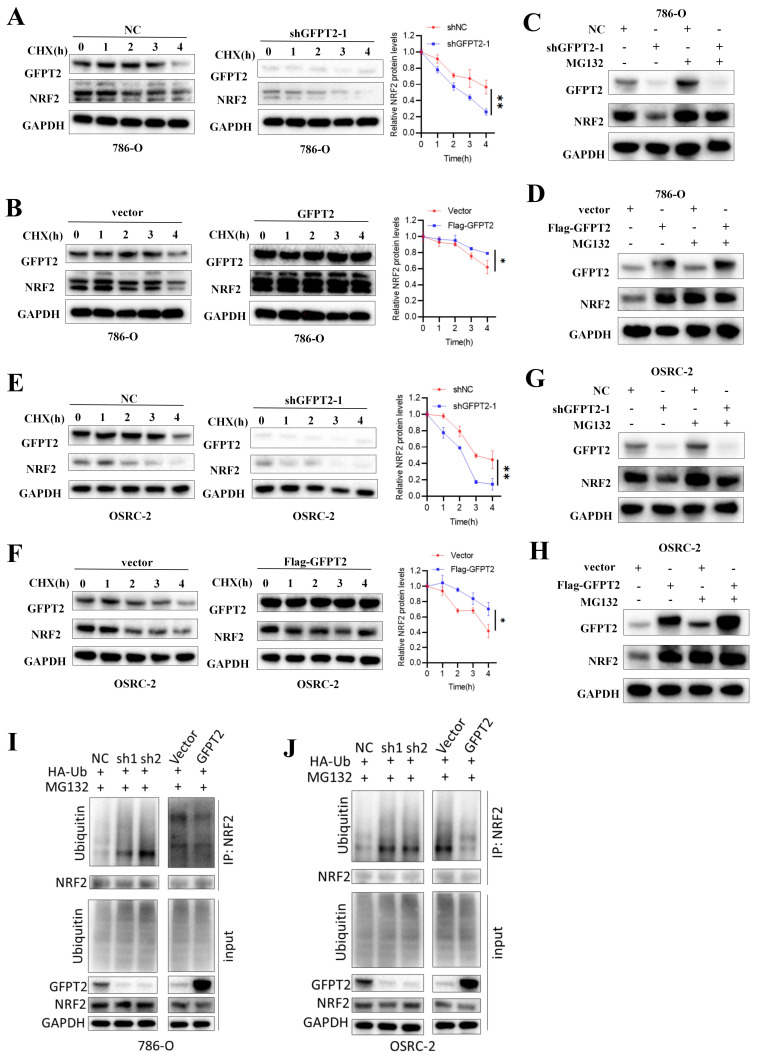
** GFPT2 impeded the degradation of NRF2.** (A-B). 786-O cells transfected with the shNC, shGFPT2, vector or GFPT2-oe were treated with CHX (10µg/ml), and collected at the indicated times for Western Blot. NRF2 were detected. Data are presented as the mean±SEM from three independent experiments. *P < 0.05 and **P < 0.01. (C-D). 786-O, 786-O shGFPT2, 786-O vector and 786-O GFPT2-oe cells were treated with or without the proteasome inhibitor MG132 (20μM,0-8h) and then NRF2 were detected. (E-F). OSRC-2 cells transfected with the shNC, shGFPT2, vector or GFPT2-oe were treated with CHX (10µg/ml), and collected at the indicated times for Western Blot. NRF2 were detected. Data are presented as the mean±SEM from three independent experiments. *P < 0.05 and **P < 0.01. (G-H). OSRC-2, OSRC-2 shGFPT2, OSRC-2 vector and OSRC-2 GFPT2-oe cells were treated with or without the proteasome inhibitor MG132 (20μM,0-8h) and then NRF2 were detected. (I-J). 786-O and OSRC-2 cells were co-transfected with the shNC, shGFPT2-1, shGFPT2-2, vector or GFPT2-oe and HA-Ub, and cell lysates were subjected to IP with NRF2 antibody, followed by IB with indicated antibodies. Cells treated with 20μM MG132 for 8h.

To determine whether GFPT2 promotes NRF2 activity by enhancing its protein stability, 786-O and OSRC-2 cells were treated with the protein synthesis inhibitor cycloheximide (CHX). We observed that GFPT2 knockdown significantly accelerated NRF2 degradation (Fig. 6A and Fig. 6E), whereas GFPT2 overexpression exerted the opposite effect (Fig. 6B and Fig. 6F).

To confirm whether NRF2 protein levels are regulated by ubiquitination and proteasome-mediated degradation, we treated 786-O and OSRC-2 cells with or without the proteasome inhibitor MG132. MG132 treatment restored NRF2 protein levels in GFPT2-knockdown cells (Fig. 6C-D and Fig. 6G-H). In addition, we found that GFPT2 knockdown significantly increased NRF2 ubiquitination in 786-O and OSRC-2 cells, while GFPT2 overexpression reduced NRF2 ubiquitination (Fig. 6I and Fig. 6J). In conclusion, we established that GFPT2 impeded the degradation of NRF2.

### GFPT2 regulates NRF2 levels in a KEAP1-binding dependent manner

No ubiquitination-related function of GFPT2 has been reported in the literature, but KEAP1-NRF2 interaction causes NRF2 degradation by ubiquitin-proteasome. We therefore investigated whether GFPT2 influences NRF2 degradation by binding to KEAP1.

**Figure 7:**
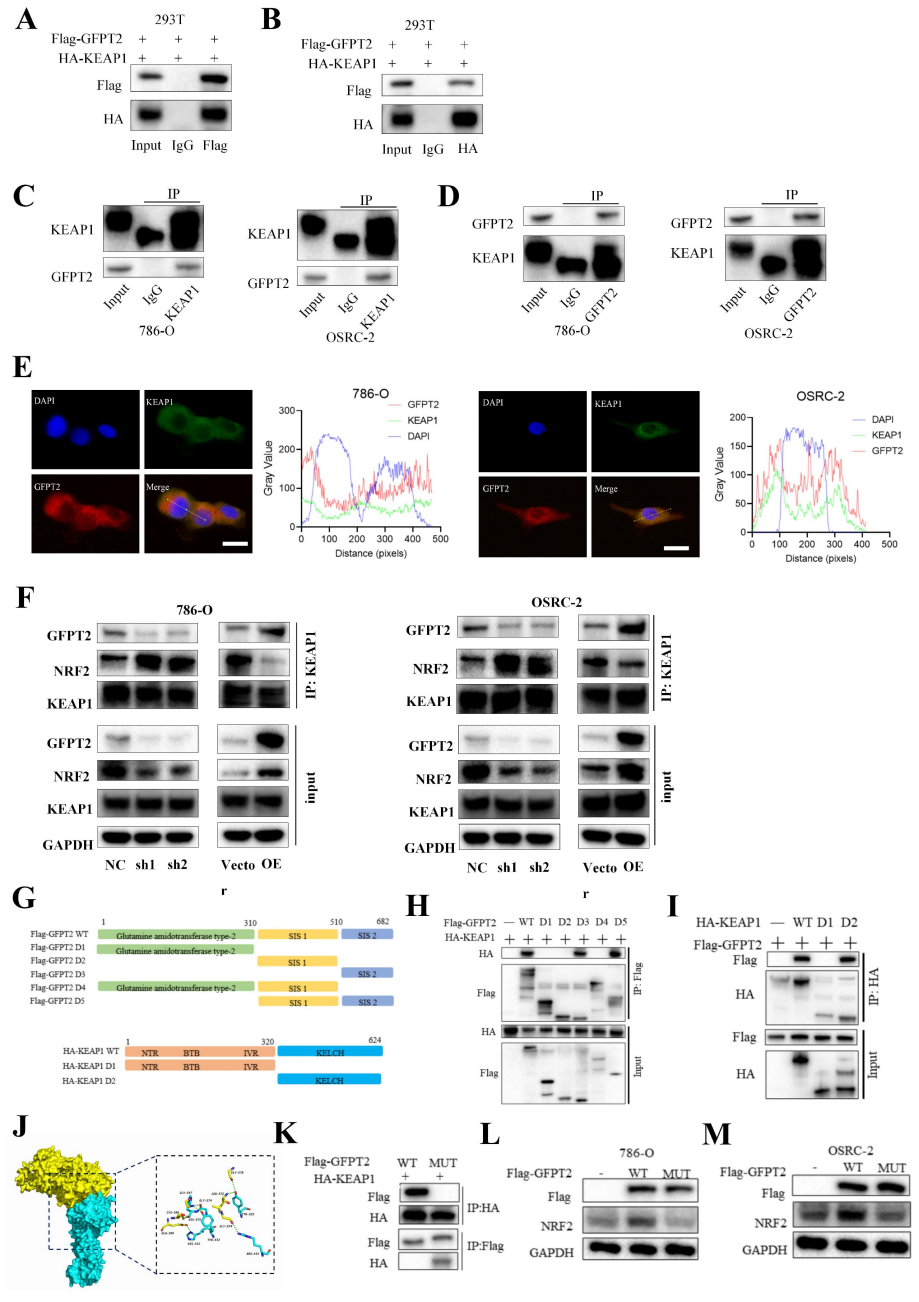
** GFPT2 regulates NRF2 levels in a KEAP1-binding dependent manner.** (A-B). HEK293T cells were transfected with Flag-GFPT2 and HA-KEAP1 alone or in combination. IP and immunoblotting analyses were performed with the indicated antibodies after 48h of transfection. (C-D). Lysates from 786-O and OSRC-2 cells were subjected to IP and immunoblotting analysis with the indicated antibodies. (E). Confocal images showing colocalization of GFPT2 (red) and KEAP1 (green) in 786-O and OSRC-2 cells. Cell nucleus was counterstained with DAPI. Scale bars, 10μm. (F). Western blot analysis of KEAP1 and NRF2 expression in shGFPT2-1 or shGFPT2-2 or GFPT2-OE 786-O and OSRC-2 cells. (G). Schematic representation of Flag-tagged full-length (FL) GFPT2, HA-tagged FL KEAP1, and their various deletion mutants. (H). HEK293T cells were co-transfected with HA-KEAP1 and Flag-tagged FL GFPT2 or its deletion mutants, and cell lysates were analyzed by IP with Flag beads followed by IB with antibodies against HA and Flag. (I). HEK293T cells were co-transfected with Flag-GFPT2 and HA-tagged FL KEAP1 or its deletion mutants, and cell lysates were analyzed by IP with HA beads followed by IB with antibodies against HA and Flag. (J). 3D interaction diagram between KEAP1 and GFPT2. (K). HEK293T cells were transfected with HA-KEAP1 and Flag-GFPT2 WT or MUT in combination. IP and immunoblotting analyses were performed with the indicated antibodies after 48h of transfection. (L-M). 786-O and OSRC-2 cells transfected with the Flag-GFPT2 WT or Flag-GFPT2 MUT were collected for western blot.

Co-immunoprecipitation (Co-IP) analysis showed that GFPT2 co-precipitated with KEAP1 in 786-O, OSRC-2, and HEK-293T cells (Fig. 7A-D), confirming the interaction between these two proteins. Given that the key function of KEAP1 is to regulate the cellular antioxidant response by interacting with NRF2, we further performed Co-IP analysis to examine the potential interaction between GFPT2 and NRF2. Endogenous NRF2 co-precipitated with endogenous KEAP1, but it failed to interact with GFPT2 alone—indicating that GFPT2 interacts exclusively with KEAP1, rather than with NRF2. Furthermore, confocal microscopy revealed that GFPT2 and KEAP1 co-localized primarily in the cytoplasm (Fig. 7E).

KEAP1 contains five conserved domains. To identify the specific domains of KEAP1 that mediate its interaction with GFPT2, we constructed a series of HA-tagged KEAP1 truncation mutants and Flag-tagged GFPT2 truncation mutants (Fig. 7G). We then co-expressed these mutants in 293T cells. As shown in Fig. 7H, the C-terminal region of GFPT2 (SIS domain, amino acids 510-682) is necessary and sufficient for direct interaction with KEAP1. Moreover, both wild-type (WT) KEAP1 (containing the full-length linker region) and KEAP1 mutants harboring a partial Kelch domain interacted with GFPT2, whereas those lacking the Kelch domain (amino acids 320-624) failed to do so (Fig. 7I).

Remarkably, the region spanning glycine (GLY) 547 to GLY 574 was predicted to be a protein-protein interaction site using the consensus neural network method for protein-protein interaction site prediction (Fig. 7J). To verify whether this GLY547-GLY574 region is the key interaction site between GFPT2 and KEAP1, we mutated the amino acids in this region to alanine in HEK-293T cells. As shown in Fig. 7K, HA-tagged KEAP1 failed to co-precipitate with Flag-tagged GFPT2 (mutant, MUT) in HEK-293T cells. Additionally, when the GFPT2-KEAP1 binding sites were mutated, overexpression of the mutant GFPT2 also failed to restore NRF2 expression (Fig. 7L-M). These data suggest that GFPT2 regulates NRF2 stability by modulating NRF2 polyubiquitination and KEAP1-mediated proteasomal degradation.

### GFPT2 regulates sunitinib resistance through enzyme-independent manners

**Figure 8:**
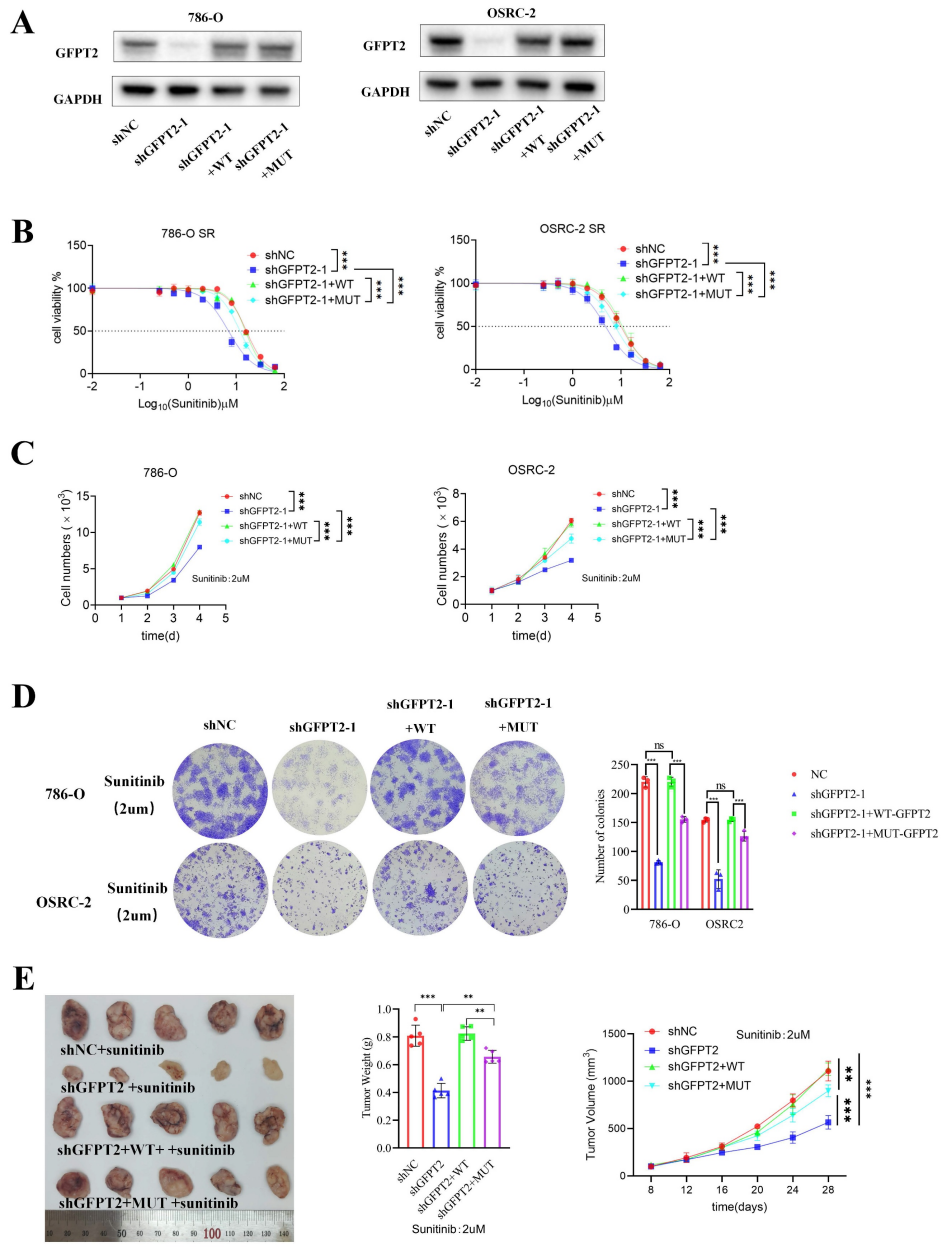
** GFPT2 regulates sunitinib resistance through enzyme-independent manners.** (A). Western blot analysis of NRF2 expression in shGFPT2-1 or shGFPT2-1+ Flag-GFPT2 WT or shGFPT2-1+ Flag-GFPT2 MUT 786-O and OSRC-2 cells. (B). 786-O and OSRC-2 cells were transfected with the shNC, shGFPT2-1 or shGFPT2-1+ Flag-GFPT2 WT or shGFPT2-1+ Flag-GFPT2 MUT. These cells were treated with a serial dose of sunitinib for 24h and subjected to CCK-8 assay. (C). 786-O and OSRC-2 cells were transfected with the shNC, shGFPT2-1 or shGFPT2-1+ Flag-GFPT2 WT or shGFPT2-1+ Flag-GFPT2 MUT. After puromycin selection, these cells were treated with or without sunitinib (2μM) for 96h and subjected to CCK-8 assay. Results are derived from three independent experiments. P values were determined by two-tailed t test. ***P < 0.001. (D). 786-O and OSRC-2 cells were transfected with the shNC, shGFPT2-1 or shGFPT2-1+ Flag-GFPT2 WT or shGFPT2-1+ Flag-GFPT2 MUT. These cells were determined using colony formation assay after sunitinib (2μM) treatment. Results are derived from three independent experiments. P values were determined by two-tailed t test. ns, not significant and ***P < 0.001. (E). OSRC-2 cells were transfected with indicated constructs for 72 h. After puromycin selection, these cells were subcutaneously injected into the nude mice. These mice were treated with or without sunitinib (oral administration, 25 mg/Kg, once a day for 8 days). The tumor image, tumor weight and tumor volume curve were shown in panel E.

We constructed wild-type (WT) and interaction site mutant (MUT) versions of GFPT2, as previously reported, and reconstituted GFPT2-WT and GFPT2-MUT in 786-O and OSRC-2 cells with endogenous GFPT2 knockdown (Fig. 8A). To assess the impact of GFPT2-MUT on modulating sunitinib sensitivity, we performed CCK-8 assays to generate sunitinib dose-response curves. GFPT2-MUT overexpression resulted in incomplete restoration of sunitinib resistance in RCC cells, whereas GFPT2-WT overexpression effectively restored this resistance (Fig. 8B).

Furthermore, GFPT2-MUT only weakly enhanced sunitinib resistance—an effect significantly less potent than that of GFPT2-WT overexpression—as confirmed by CCK-8 assays, colony formation assays, and in vivo experiments (Fig. 8C-E). In summary, these findings indicate that GFPT2 regulates NRF2 through non-metabolic interaction with KEAP1, thereby promoting sunitinib resistance in RCC cells.

## Discussion

Metabolic reprogramming plays an important role in tumor proliferation and metastasis, and amino acid metabolic reprogramming provides energy for the unrestricted proliferation and metastasis of malignant tumors. In addition to increasing metabolic flux, several metabolic enzymes have recently been reported to have discovered functions other than metabolism, playing important roles in cell cycle regulation, apoptosis control, and tumor cell proliferation and metastasis 42, 43. In this paper, we confirmed that GFPT2, a key enzyme in the hexosamine synthesis pathway, plays a role in regulating sunitinib sensitivity of renal cancer. GFPT2, an enzymatic entity intricately linked to glutamine metabolism, was identified as a regulator of the glutamine metabolic cascade into the Hexosamine Biosynthetic Pathway (HBP). Through the modulation of HBP flux, GFPT2 orchestrated the O-GlcNAcylation of Yes-associated protein (YAP1), facilitating its translocation into the cellular nucleus. Subsequently, YAP1 exercised its transcriptional regulatory authority over downstream genes, thereby instigating Sunitinib resistance. Additionally, the study uncovered an ancillary regulatory role of GFPT2 in NRF2.

Previous reports have shown that glutamine plays an important role in tumor cell proliferation 44-46. Analysis of some Sunitinib resistance model data shows that glutamine metabolism plays a role in drug resistance, but the specific mechanism is not clear. We experimentally confirmed that glutamine deprivation inhibits the proliferation of renal cancer cells, and the proliferation of Sunitinib-resistant cells is more dependent on exogenous glutamine uptake. We found that GFPT2 is a key protein in glutamine metabolism that plays a role in Sunitinib resistance.

GFPT2 plays a context-specific role in cancer progression, with remarkably distinct implications in renal cell carcinoma as opposed to other types of malignant tumors. In RCC, GFPT2 overexpression has been linked to sunitinib resistance by enhancing hexosamine biosynthesis pathway (HBP) flux and O-GlcNAcylation. In contrast, in colorectal cancer, GFPT2 promotes metastasis via O-GlcNAcylation of p65, forming a positive feedback loop 25, while in pancreatic cancer, it drives macrophage M2 polarization to foster immune evasion 47. These differences highlight tissue-specific metabolic reprogramming and underscore GFPT2 as a versatile therapeutic target. We also confirmed that OGT, a key enzyme mediating O-GlcNAcylation, also promotes the proliferation and metastasis of renal cancer. Inhibition of OGT enzyme activity can restore sunitinib sensitivity. However, to address the question of how glycosylation regulates downstream proteins to exert their catalytic functions, we examined the role of the previously reported downstream YAP1 protein—and our findings were consistent with those of previous studies.

Some recent studies have found that metabolic enzymes have part-time functions in addition to regulating metabolism 48. Our study confirmed that the therapeutic effect of YAP1 knockdown in coordination with Sunitinib is weaker than that of GFPT2 knockdown in coordination with Sunitinib, indicating that the metabolic function of GFPT2 cannot fully explain the increased sunitinib sensitivity caused by enzymatic pathway, which suggests that we should seek for a potential mechanism. By transcriptome sequencing analysis, we found that GFPT2 knockdown caused the down-regulation of antioxidant genes, which was contrary to the results reported in the literature 24. GFPT2 regulates glycosylation to promote drug resistance, but the increase of glycosylation level leads to the degradation of NRF2, which cannot reasonably explain the difference in NRF2 expression. Our results confirm that GFPT2 regulates NRF2 in a more direct way, that is, it inhibits NRF2 ubiquitination after binding with KEAP1, thereby up-regulating NRF2 expression. KEAP1 continues to ubiquitinate NRF2 under physiological conditions, keeping it at a low level. When NRF2 dissociates from KEAP1 and enters the nucleus after electrophilic stimulation or oxidative stress, NRF2 and KEAP1 transcribe HMOX1 together with the ARE binding box, NQO1 plays an antioxidant role. Therefore, NRF2 has been found in many studies to cause insensitivity to tumor drug therapy by reducing ROS levels 49, 50. We demonstrated that GFPT2 competitively binds to the KLECH domain of KEAP1, resulting in reduced NRF2 binding and ubiquitination, which plays a role in promoting the development of drug resistance. By constructing mutant plasmid analysis, we confirmed that the recovery of proliferation and drug sensitivity of MUT plasmid transfected with GFPT2 knockdown cannot be exactly the same as that of WT plasmid, indicating that the part-time function of GFPT2 only plays a partial role, and metabolic function still plays a role in drug resistance.

Thus, the identification of GFPT2 as a key mediator of sunitinib resistance through metabolic reprogramming opens promising therapeutic avenues. Building upon current RCC treatment paradigms that combine antiangiogenic agents with immunotherapy, our findings suggest that GFPT2 inhibition could synergize with existing standard-of-care regimens. Specifically, targeting the GFPT2-HBP axis may reverse the immunosuppressive tumor microenvironment by reducing M2 macrophage polarization and T-cell exhaustion markers, thereby potentially enhancing the efficacy of immune checkpoint inhibitors 47, 51, 52. This approach aligns with the observed clinical benefits of TKI-ICI combinations in RCC, while addressing metabolic resistance mechanisms that limit durable responses.

However, challenges remain. GFPT2's metabolic plasticity may lead to compensatory activation of alternative pathways (e.g., glycolysis). The lack of selective GFPT2 inhibitors necessitates development of isoform-specific drugs. Future studies should explore nanoparticle-delivered GFPT2 siRNA or allosteric inhibitors to improve specificity and efficacy. Collectively, while GFPT2-targeted strategies offer a rational approach to combat sunitinib resistance, their success hinges on overcoming metabolic adaptability and toxicity hurdles.

Finally, our study still has some limitations. We did not confirm how glutamine affects GPFT2 expression levels, which may be a potential mechanism to target. Inhibiting GFPT2 expression can inhibit both glycosylation and oxidative stress, but because o-glycosylation modification is too extensive, we believe that developing inhibitors that target GFPT2 binding to KEAP1 may have lower side effects. The effect of GFPT2 knockdown on glycosylation does not seem to be as significant as that of direct OGT inhibition, which may be due to the negative feedback inhibition of GFPT2 enzyme activity by O-GlcNAcylation level. Several studies have demonstrated that elevated UDP-GlcNAc levels can inhibit GFPT2 enzymatic activity 53, creating a compensatory mechanism that limits the impact of GFPT2 knockdown. This feedback regulation contrasts with direct OGT inhibition, which bypasses this metabolic control and more effectively reduces O-GlcNAcylation. Additionally, due to the similar enzymatic activities shared by GFPT1 and GFPT2, functional redundancy with GFPT1 and OGT's preferential modification of critical substrates may further diminish the effect of GFPT2 knockdown 54, 55. These findings suggest that targeting OGT directly may be more effective than modulating GFPT2 activity for reducing protein O-GlcNAcylation in cellular systems. In addition, this study still has a notable limitation: whether GFPT2 exerts this effect by interfering with the recruitment of Cul3 E3 ligase to KEAP1—a key step in the KEAP1-mediated NRF2 degradation pathway. Although our Co-IP data demonstrated that GFPT2 specifically binds to the Kelch domain of KEAP1 rather than the N-terminal Cul3-binding domain (CBD), we have not yet directly verified the impact of GFPT2 on the KEAP1-Cul3 interaction using a KEAP1 mutant lacking the Cul3-binding domain (KEAP1-ΔCBD). This unresolved issue will be thoroughly addressed in our subsequent studies.

Our study expands researchers' understanding of the function of metabolic enzymes, and in future studies, more metabolic enzymes may be found to have part-time functions outside metabolism, and drugs developed by targeting activities outside of the enzyme's activity may be more advantageous.

## Materials and Methods

### Clinical samples and database

We obtained transcriptional and clinical data from the official website of the Cancer Genome Atlas (TCGA). All patients with primary RCC (each pair was from the same patient) were collected from the Department of Urology of the First Affiliated Hospital of Nanjing Medical University at the time of operation. All tumor collection and analysis were approved by the Ethics Committee of Nanjing Medical University. All patients received informed consent. The histological features of the tissue were examined independently by two urologists in accordance with WHO standards. The clinicopathological characteristics of these patients have been Supplementary Tables 1.

### Cell lines and cell culture

The human renal cell carcinoma cell lines 786-O and OSRC-2 were purchased from Procell Life Science & Technology (Wuhan, China). 786-O cells and OSRC-2 cells were cultured in complete media containing 1% P/S and 10% FBS. All cells were incubated at 5%CO2 and 37 ° C. All cells were identified by STR and tested for mycoplasma every 6 months. Sunitinib-resistant cells were cultured from low concentration to high concentration according to previous methods, and IC50 was detected after 20 generations of stable passage in 10uM Sunitinib, indicating that drug-resistant cells were successfully constructed. Information on use of medications was shown in Supplementary Table 2.

### Transfection of shRNA and plasmids

Lentiviral vectors encoding target genes, short hairpin RNAs (shRNAs), and empty vectors were synthesized by Genechem (Shanghai, China). Cells were placed in a six-well plate, then 5ng/ml of viral transfection agent polybrene was added with appropriate amount of disease venom, the fluid was changed 6 hours later, and 5ug/ml of purinomycin was added 48 hours later for screening, the fluid was changed 24-48 hours later, and cells were cultured with a medium containing 2ug/ml of purinomycin.

Flag-GFPT2 (WT, D1, D2, D3, D4, D5), HA-KEAP1 (WT, D1, D2), Myc-NRF2 plasmids were purchased from Changsha Yoose Biotechnology. Cell transfection was performed according to lipo3000 instructions, and cells were collected 48 hours later for follow-up experiments.

### Cell proliferation assay

After cell counting, 1000 cells per 100ul medium per hole in the 96-well plate were detected at 24, 48, 72 and 96 hours. CCK8 reagents are purchased from apex. Before the test, fresh medium was used, and 10ul cck8 reagent was added to 100ul per empty space. 1.5 hours later, OD value was detected at A450 wavelength with enzyme marker. 200ul PBS was added around the 96-well plate to prevent evaporation of intermediate medium from affecting the results. IC50 means that the value is fitted to the curve with software to calculate the IC50 value. The IC50 values have been shown in Supplementary Table S5.

### Colony formation experiment

There were 1000 cells in each well of the 6-well plate. After 7-10 days, the medium was abandoned, and then fixed with paraformaldehyde for 15-30 minutes after washing with PBS. After washing with PBS, crystal violet was stained for 15-30 minutes.

### Real-time fluorescence quantitative (PCR)

Cell RNA was extracted according to Vazyme total RNA extraction kit, reverse transcription was performed according to Vazyme R333 kit, and 10ul system was configured according to vazyme R341 kit. The RCHO machine was mounted according to R341 temperature and time. Primers are described in Supplementary Table 3.

### RNA-sequencing and data analysis

Total RNA was extracted from clear cell renal cell carcinoma (ccRCC) cells using TRIzol® Reagent (Magen), per the manufacturer's standard protocol. For RNA quality evaluation, the A260/A280 absorbance ratio was measured via a Nanodrop ND-2000 spectrophotometer (Thermo Scientific, USA), while the RNA Integrity Number (RIN) was evaluated using an Agilent Bioanalyzer 4150 system (Agilent Technologies, CA, USA).

Paired-end RNA sequencing libraries were constructed with the ABclonal mRNA-seq Library Preparation Kit (ABclonal, China) as per the manufacturer's guidelines. High-throughput sequencing was conducted on either the Illumina NovaSeq 6000 or MGISEQ-T7 platform.

Sequencing data generated by Illumina or BGI platforms were utilized for downstream bioinformatics analyses. Differential gene expression analysis was carried out using the DESeq2 R package (available at http://bioconductor.org/packages/release/bioc/html/DESeq2.html). Genes with an absolute log2 fold change (|log2FC|) > 1 and a P-value < 0.05 were defined as significantly differentially expressed genes (DEGs).

The clusterProfiler R package was employed to conduct Gene Ontology (GO) functional enrichment and Kyoto Encyclopedia of Genes and Genomes (KEGG) pathway enrichment analyses. A P-value < 0.05 served as the threshold to identify significantly enriched GO terms or KEGG pathways.

### Western blot and immunoprecipitation (IP)

Cells or tissue samples of interest were harvested and lysed on ice in cell lysis buffer supplemented with protease inhibitors to prepare total protein extracts containing the target protein. Cellular debris was then cleared by centrifugation at 4°C. Subsequently, the supernatant was fully incubated with a target protein-specific antibody (usually overnight at 4°C) to facilitate specific antibody-target protein binding. After this step, Protein A/G agarose beads (or magnetic beads) were added, and incubation proceeded at 4°C for 12 hours. This allowed the agarose beads to capture the complex by binding to the antibody's Fc region. Post-incubation, the precipitate was collected via low-temperature centrifugation and gently washed several times with washing buffer to eliminate non-specifically bound proteins. Finally, SDS-PAGE loading buffer was added to the precipitate, and the mixture was boiled to dissociate the protein complex from the agarose beads. The supernatant was collected by centrifugation, and proteins were separated using SDS-PAGE electrophoresis, followed by Western blotting to detect the target protein and its co-precipitated interacting proteins. Antibody information is provided in Supplementary Table S4.

### Immunohistochemistry (IHC)

Formalin-fixed paraffin-embedded (FFPE) tissue sections (4-5 μm thick) were processed for IHC staining using a modified standard protocol. Briefly, sections were dewaxed sequentially in xylene (three exchanges, 10 minutes each) and rehydrated via a graded ethanol gradient (100%, 95%, 85%, 70%, 5 minutes per concentration), followed by rinsing with double-distilled water (ddH₂O). ntigen retrieval was achieved by boiling the sections in 10 mM citrate buffer (pH 6.0) using a 750-W microwave oven for 15 minutes; sections were then allowed to cool naturally to room temperature (RT) for 30 minutes. After cooling, sections were rinsed three times (5 minutes each) with phosphate-buffered saline (PBS, pH 7.4) containing 0.05% Tween-20 (PBST). To block non-specific antibody binding, sections were incubated with 5% bovine serum albumin (BSA) dissolved in PBST at RT for 60 minutes. Primary antibodies were added dropwise onto the sections, which were then placed in a humidified chamber and incubated at 4°C overnight. Post-primary antibody incubation, sections were washed three times (5 minutes each) with PBST and then incubated with horseradish peroxidase (HRP)-conjugated secondary antibody at RT for 45 minutes. Following three additional PBST washes (5 minutes each), immunoreactive signals were visualized with 3,3'-diaminobenzidine (DAB) chromogen solution. Color development was monitored under a light microscope and halted after 5-8 minutes—before non-specific background staining emerged. Sections were counterstained with hematoxylin for 2 minutes, differentiated in 1% hydrochloric acid-ethanol solution for 30 seconds, and blued in 0.5% ammonia water for 1 minute. Finally, sections were dehydrated using a graded ethanol gradient (70%, 85%, 95%, 100%, 5 minutes per concentration), cleared with xylene (three exchanges, 10 minutes each), and mounted with neutral balsam. All staining steps were conducted under uniform conditions, and tissue images were acquired using a digital pathology slide scanner.

### Immunofluorescence (IF)

The sterile cell climbing tablets were placed in 24-well plates, 5000-10000 cells were added to each well, and the medium was discarded 24 hours after treatment, washed with PBS, fixed with formaldehyde for 30 minutes, washed with PBS, 0.3% TritonX100 permeable for 10 minutes, washed with PBS shaker for 5 minutes, and sealed with Beyotime immunostaining blocking solution for 15-30 minutes. After finishing, dilute the first antibody with Beyotime immunostaining diluent according to the instructions, cover the crawling tablets with 50-100ul antibodies per well, overnight at 4°C or at room temperature for 2 hours without shaking, shake PBST for 5 minutes after finishing, dilute the second antibody with immunofluorescence staining diluent of the second antibody, avoid light for incubation of the second antibody and subsequent experiments, and PBS should be used at room temperature for 1 hour.

### Apoptosis detection

After cell treatment, the cells were digested in an incubator with Gibco EDTA-free pancreatic enzyme at 37°C for 30 minutes, and terminated after the cells were completely digested. Do not blow the cells to avoid excessive influence on the results of necrotic cells. The cells were treated and stained with apexbio apoptosis kit, and the results were analyzed with Beckmann depletion cytometer.

### Xenotransplantation model

The 4-week-old nude mice were purchased from Charles River and fed in the Animal Experimental Center of Nanjing Medical University in a suitable environment. The experiment started 1-2 weeks later. Kidney cancer OSRC-2 cell suspension was injected into the midline of the abdomen of each mouse near the forelimb, and 5*10^6^ cells were mixed with 100ul PBS+100ul ABW matrix glue and injected into the subcutaneous of the mouse. Observations were made every 4 days, when the tumor size was measured after 50 mm^3^, and the volume was calculated in terms of major axis * minor axis * minor axis /2. Starting from day 8, control group and treatment group were divided into 2 groups, one group was given Sunitinib 25mg/kg gavage for 8 consecutive days. The experiment was terminated when the long axis was < 15mm or the volume was < 1000 mm^3.^ The tumor volume, tumor weight, and weight of mice before and after were measured, and the growth curve was described for statistical analysis. All experiments were carried out with the approval of the Animal Ethics Committee of Nanjing Medical University.

### Statistics

With reference to other literature, experimental data are shown as mean ± standard deviation, and the number of experimental data is shown in the paper. GranhPad8.0 was used for statistical analysis. The data of 2 groups were analyzed by bilateral T test, and the significant differences of more than 2 groups were calculated by one-way ANOVA. P< 0.05 marked with *, P< 0.01 marked as **, P< 0.001 marked as ***, P< 0.0001 marked as ****. No significant difference was marked as ns.

## Supplementary Material

Supplementary figures and tables.

## Data Availability

All data needed to evaluate the conclusions in the paper are present in the main text or the Supplementary Materials. Additional data related to this paper may be requested from the authors.
