# Supplementary material for: GFPT2 drives sunitinib resistance of renal cell carcinoma via enzyme-dependent and -independent manners
Source: Int J Biol Sci. 2026 Feb 4;22(5):2324–42. doi: 10.7150/ijbs.118985 (PMC12965143; doi:10.7150/ijbs.118985)
Supplement: Supplementary file 1 — Supplementary figures and tables. [file ijbsv22p2324s1.pdf]

Supplementary Materials for

GFPT2 drives sunitinib resistance of clear cell renal cell carcinoma via enzyme-dependent and -independent manners

Authors

Songbo Wang[1,a], Jiajun Xing[1,a], Xiaoyi Wang[2,a], Zengjun Wang[1,*], Pengfei Shao[1,*], Chenkui Miao[1,*]

Affiliations

[1] Department of Urology, The First Affiliated Hospital of Nanjing Medical University, Nanjing, China
[2] Core Facility Center, The First Affiliated Hospital of Nanjing Medical University, Nanjing, China

[*]**Corresponding Author.** Email: zengjunwang@njmu.edu.cn, spf8629@163.com, medicalmck@163.com

[a]These authors contributed equally to this work.

This PDF file includes:

Figs. S1 to S4
Tables S1 to S5

Supplemental Figures

Fig. S1.

(A). Optical micrograph showing 786-O and OSRC-2 cells with sunitinib resistance (786-O SR and OSRC-2 SR).

(B). 786-O, OSRC-2, 786-O SR and OSRC-2 SR cells were with a serial dose of sunitinib for 24h and subjected to CCK-8 assay.

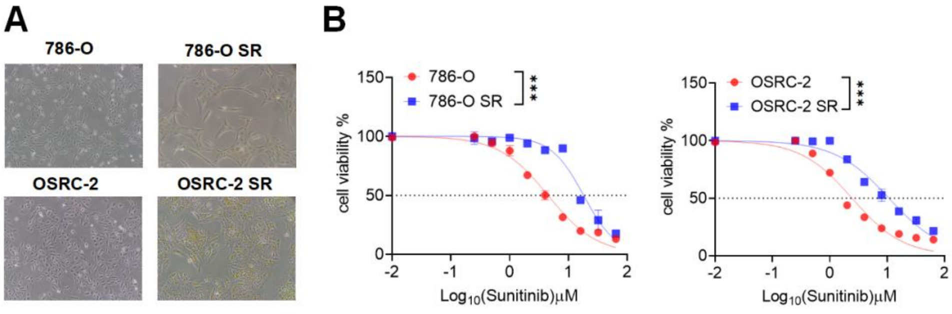

A
786-O
786-O SR
OSRC-2
OSRC-2 SR
B
786-O
786-O SR
***
cell viability %
Log10(Sunitinib)μM
OSRC-2
OSRC-2 SR
***
cell viability %
Log10(Sunitinib)μM

Fig. S2.

(A). mRNA expression levels of GFPT2 in various RCC cell lines from the CCLE database.

(B). GFPT2 mRNA levels in normal and tumor tissues from the TCGA cohort.

(C). Kaplan–Meier survival plots of OS according to GFPT2 mRNA expression in RCC specimens from the TCGA RCC cohort.

(D). Relationship between GFPT2 mRNA expression and T stage, pathological TNM stage in patients in the TCGA RCC cohort.

(E). qRT-PCR verification of GFPT2 mRNA expression between RCC cell lines.

(F). WB detected GFPT2 protein expression in RCC cell lines.

(G). WB detected GFPT2 protein expression between RCC tissues (T) and adjacent noncancerous tissues (N) from the NJMU RCC cohort. The protein level of GFPT2 relative to that of actin was measured using ImageJ.

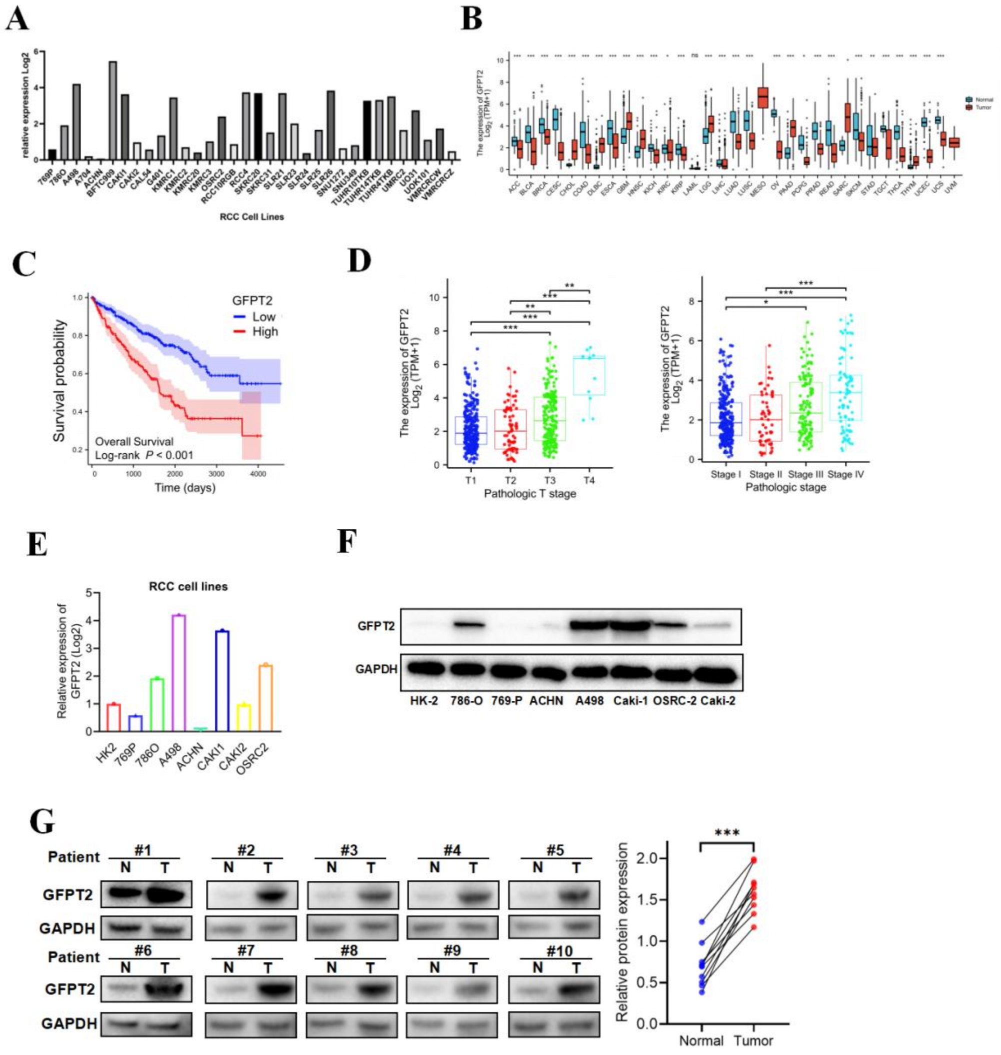
A
relative expression Log2
RCC Cell Lines
B
The expression of GFPT2 Log2 (TPM+1)
Normal
Tumor
C
GFPT2
Low
High
Survival probability
Overall Survival
Log-rank P < 0.001
Time (days)
D
The expression of GFPT2 Log2 (TPM+1)
T1
T2
T3
T4
Pathologic T stage
Stage I
Stage II
Stage III
Stage IV
Pathologic stage
E
RCC cell lines
Relative expression of GFPT2 (Log2)
HK2
769P
786O
A498
ACHN
CAKI1
CAKI2
OSRC2
F
GFPT2
GAPDH
HK-2
786-O
769-P
ACHN
A498
Caki-1
OSRC-2
Caki-2
G
Patient
#1
#2
#3
#4
#5
#6
#7
#8
#9
#10
N
T
GFPT2
GAPDH
Relative protein expression
Normal
Tumor
***

Fig. S3.

(A). 786-O and OSRC-2 cells were transfected with indicated constructs for 24 h. Cells were treated with or without sunitinib (2 μM) for another 24 h. Cells were collected for Caspase 3 activity assay. Data presents as mean ± SEM with three replicates. ns, not significant, *P < 0.05, **P < 0.01 and *** P < 0.001.

(B). Tumor volumes and tumor weight were measured in the indicated groups. Data presents as mean±SEM with five replicates. *P <0.05; ***P <0.001.

(C). Representative images of HE and IHC for Ki-67. Scale bar, 50μm.

(D). 786-O and OSRC-2 cells were transfected with or without Flag-GFPT2. These cells were treated with a serial dose of sunitinib for 24h and subjected to CCK-8 assay. The IC50 values of sunitinib in each group were indicated.

(E-G). 786-O and OSRC-2 cells were transfected with indicated constructs for 48 h. After 24 h puromycin selection, cells were treated with or without sunitinib (2μM) for another 24 h. Cells were collected for Annexin v-FITC/7-AAD assay(F), Western blot analysis(F) and Caspase 3 activity assay(G). Data presents as mean ± SEM with three replicates. Ns, not significant and **P < 0.01.

(H). 786-O and OSRC-2 cells were transfected with indicated constructs for 72 h. After puromycin selection, these cells were treated with or without sunitinib (2μM) for 96h and subjected to CCK-8 assay. Data presents as mean ± SEM with three replicates. P values were determined by two-tailed t test. ***P < 0.001.

(I). The growth of 786-O, OSRC-2, 786-O Flag-GFPT2 and OSRC-2 Flag-GFPT2 cells were determined using colony formation assay after sunitinib (2μM) treatment. Data presents as mean ± SEM with three replicates. *** $P < 0.001$.

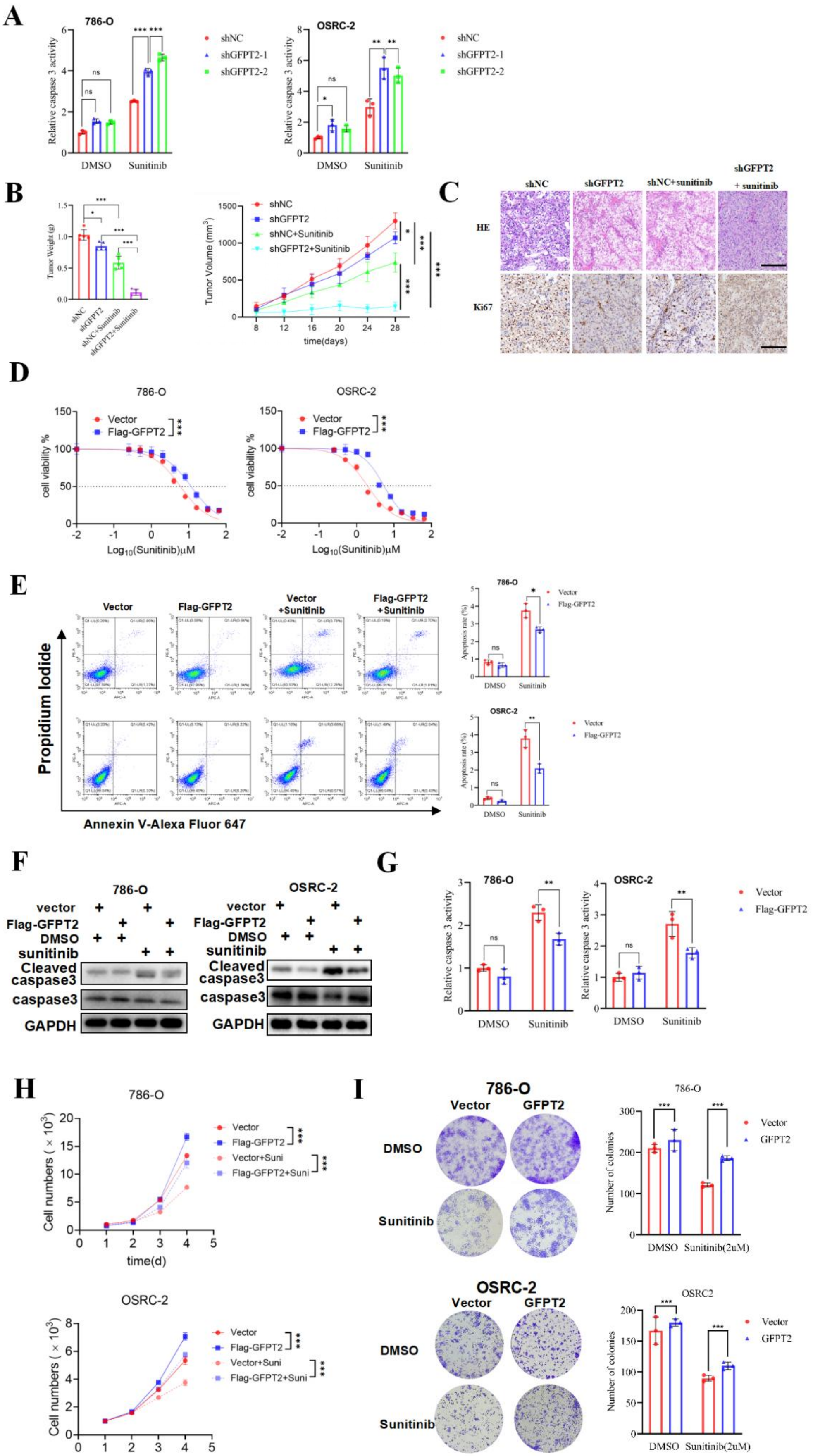
A
786-O
OSRC-2
shNC
shGFPT2-1
shGFPT2-2
Relative caspase 3 activity
DMSO
Sunitinib
B
C
shNC
shGFPT2
shNC+Sunitinib
shGFPT2+Sunitinib
Tumor Weight (g)
Tumor Volume (mm3)
time(days)
HE
Ki67
D
Vector
Flag-GFPT2
cell viability %
Log10(Sunitinib)μM
E
Vector
Flag-GFPT2
Vector +Sunitinib
Flag-GFPT2 +Sunitinib
Propidium Iodide
Annexin V-Alexa Fluor 647
Apoptosis rate (%)
F
vector
Flag-GFPT2
DMSO
sunitinib
Cleaved caspase3
caspase3
GAPDH
G
H
Vector+Suni
Flag-GFPT2+Suni
Cell numbers (×10³)
time(d)
I
GFPT2
Number of colonies
Sunitinib(2uM)

Fig. S4.

(A). HMOX1 mRNA levels in normal and tumor tissues from the TCGA cohort.

(B-D). 786-O, OSRC-2, 786-O SR and OSRC-2 SR cells were transfected with indicated constructs for 72 h. After puromycin selection, these cells were treated with a serial dose of sunitinib for 24h and subjected to CCK-8 assay. The IC50 values of sunitinib in each group were indicated.

(E). OSRC-2 cells were transfected with indicated constructs for 72 h. After puromycin selection, these cells were subcutaneously injected into the nude mice. These mice were treated with or without sunitinib (oral administration, 25 mg/Kg, once a day for 8 days). The tumor image was showed in panel E. Tumor volumes and tumor weight were measured in the indicated groups. Data presents as mean ± SEM with five replicates. *$P < 0.05$; ***$P < 0.001$.

(F). Correlation analysis of GFPT2 and NRF2 in the NJMU-ccRCC cohort (Spearman correlation, $P < 0.05$).

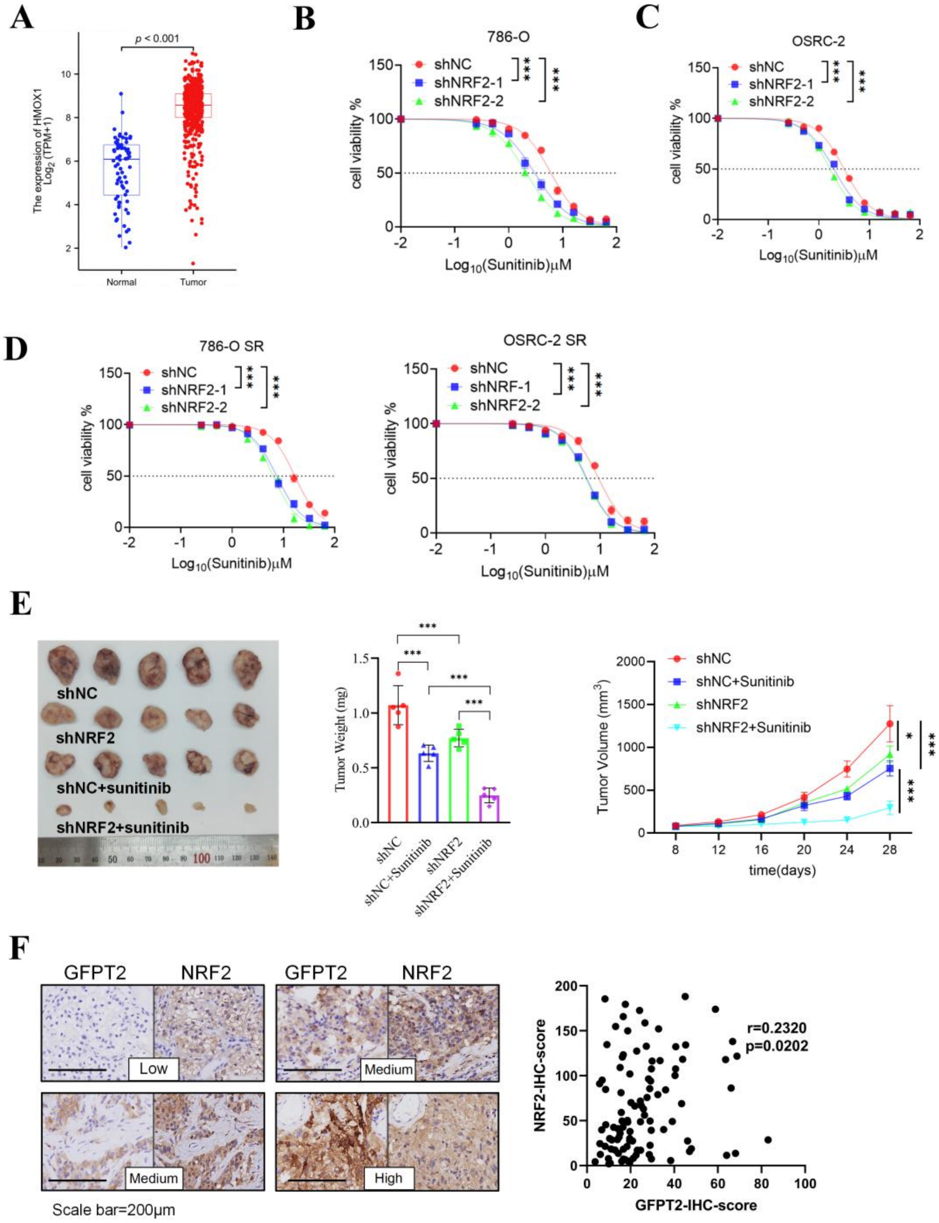
A
p < 0.001
The expression of HMOX1 Log2 (TPM+1)
Normal
Tumor
B
786-O
shNC
shNRF2-1
shNRF2-2
cell viability %
Log10(Sunitinib)μM
C
OSRC-2
D
786-O SR
OSRC-2 SR
shNRF-1
E
shNC
shNRF2
shNC+sunitinib
shNRF2+sunitinib
Tumor Weight (mg)
shNC+Sunitinib
shNRF2+Sunitinib
Tumor Volume (mm3)
time(days)
F
GFPT2
NRF2
Low
Medium
High
Scale bar=200μm
r=0.2320
p=0.0202
NRF2-IHC-score
GFPT2-IHC-score

Supplemental Tables

Table S1.

Clinical information on the tissue microarray.

	NJMU cohort
Location	
Left	51
Right	52
WHOISUP	
1	2
2	55
3	40
4	6
Size	
>4cm	80
<4cm	23
TMN(T)	
T1	54
T2	11
T3	32
T4	6

TMN(N)	
N0	102
N1	1
TMN(M)	
M0	103
M1	0

Table S2.

Sequences of small interference RNAs.

siRNA	sequences
siGFPT2-1	ACCATCGCCAAGCTGATTAAA
siGFPT2-2	ATCCGTGGCTTGAGATCTTTA
siGFPT2-3	AGGTAACTTCAGTGCGTTTAT
siNRF2-1	CCGGCAUUUCACUAAACACAA
siNRF2-2	GCAGUUCAAUGAAGCUCAACU
siNRF2-3	CAGUCUUCAUUGCUACUAAUC

Table S3.

The primer sequences for each primer set.

primers	sequences
GFPT2-F	ATGTGCGGAATCTTTGCCTAC
GFPT2-R	ATCGAGAGCCTTGACTTTCCC
GAPDH-F	GGAGCGAGATCCCTCCAAAAT
GAPDH-R	GGCTGTTGTCATACTTCTCATGG
ANKRD1-F	GTGCTGAGCAACTTATCTCGG
ANKRD1-R	CGTGGAGGAAACCTGGATGTT
CCN1-F	CGCCGAAGTTGCATTCCAG
CCN1-R	CTCGCCTTAGTCGTCACCC
CCN2-F	AACCACGGTTTGGTCCTTGG
CCN2-R	CAGCATGGACGTTCGTCTG
AKR1C1-F	TCCAGTGTCTGTAAAGCCAGG
AKR1C1-R	CCAGCAGTTTTCTCTGGTTGAA
HMOX1-F	AAGACTGCGTTCCTGCTCAAC
HMOX1-R	AAAGCCCTACAGCAACTGTCG

Table S4.

Reagent and supplier information.

Antibodies	Source	Identifier
Anti-GFPT2	Abcam	Cat#ab190966
Anti-GAPDH	Cell Signaling Technology	Cat#5174
Anti-Cleaved Caspase3	ORIGENE	Cat#TA327916S
Anti-Caspase3	ORIGENE	Cat#TA322764S
Anti-O-GlcNAc	Abcam	Cat#ab2739
Anti- OGT	Abcam	Cat#96718
Anti-YAP1	Cell Signaling Technology	Cat#14074
Anti-LaminB1	Proteintech	Cat# 12987-1-AP
Anti-HMOX1	Proteintech	Cat# 10701-1-AP
Anti-KEAP1	Proteintech	Cat#10503-2-AP
Anti-NRF2	Cell Signaling Technology	Cat#12721
Anti-NRF2	Santa Cruz	Cat#sc722
Anti-Ubiquitin	Cell Signaling Technology	Cat#3936
Anti-Flag	Cell Signaling Technology	Cat#8146
Anti-HA	Cell Signaling Technology	Cat#3724
Anti-Myc	Cell Signaling Technology	Cat#2276

Table S5.

Sunitinib IC50 in Renal Cancer Cells

Cell	Group	IC50	Cell	Group	IC50
786-O	Gln+	4.312	OSRC-2	Gln+	2.454
	Gln-	2.671		Gln-	1.289
786-O SR	Gln+	14.46	OSRC-2 SR	Gln+	8.799
	Gln-	2.547		Gln-	2.404
786-O	shNC	4.48	OSRC-2	shNC	3.601
	shGFPT2-1	1.068		shGFPT2-1	1.123
	shGFPT2-2	1.251		shGFPT2-2	0.773
786-O SR	shNC	11.73	OSRC-2 SR	shNC	8.065
	shGFPT2-1	1.808		shGFPT2-1	2.685
	shGFPT2-2	2.364		shGFPT2-2	2.411
786-O	Vector	5.96	OSRC-2	Vector	1.943
	Flag-GFPT2	11.45		Flag-GFPT2	5.353
786-O	shNC	4.801	OSRC-2	shNC	3.776
	si-YAP1	2.794		si-YAP1	2.129
	shGFPT2-1+si-YAP1	1.178		shGFPT2-1+si-YAP1	0.8191
	shGFPT2-1	1.176		shGFPT2-1	0.7176
786-O SR	shNC	16.23	OSRC-2 SR	shNC	9.943
	shGFPT2-1	6.874		shGFPT2-1	4.764
	shGFPT2-1+WT-GFPT2	15.54		shGFPT2-1+WT-GFPT2	10.64
	shGFPT2-1+MUT-GFPT2	11.96		shGFPT2-1+MUT-GFPT2	7.546
786-O	NC	4.785	OSRC-2	NC	2.43
	SR	18.08		SR	10.69
786-O	shNC	6.041	OSRC-2	shNC	3.154
	shNRF2-1	3.027		shNRF2-1	1.979
	shNRF2-2	2.115		shNRF2-2	1.666
786-O SR	shNC	16.62	OSRC-2 SR	shNC	9.36
	shNRF2-1	7.37		shNRF2-1	5.683
	shNRF2-2	6.319		shNRF2-2	5.475
